# Natural climate solutions versus bioenergy: Can carbon benefits of natural succession compete with bioenergy from short rotation coppice?

**DOI:** 10.1111/gcbb.12626

**Published:** 2019-06-13

**Authors:** Gerald Kalt, Andreas Mayer, Michaela C. Theurl, Christian Lauk, Karl‐Heinz Erb, Helmut Haberl

**Affiliations:** ^1^ Institute of Social Ecology (SEC), Department of Economics and Social Sciences University of Natural Resources & Life Sciences Vienna (BOKU) Austria

**Keywords:** bioenergy, carbon accounting, carbon sequestration, carbon stock change, climate change mitigation, CO_2_, energy plantations, land use, land‐use change, natural climate solution, natural succession, reforestation, short rotation coppice

## Abstract

Short rotation plantations are often considered as holding vast potentials for future global bioenergy supply. In contrast to raising biomass harvests in forests, purpose‐grown biomass does not interfere with forest carbon (C) stocks. Provided that agricultural land can be diverted from food and feed production without impairing food security, energy plantations on current agricultural land appear as a beneficial option in terms of renewable, climate‐friendly energy supply. However, instead of supporting energy plantations, land could also be devoted to natural succession. It then acts as a long‐term C sink which also results in C benefits. We here compare the sink strength of natural succession on arable land with the C saving effects of bioenergy from plantations. Using geographically explicit data on global cropland distribution among climate and ecological zones, regionally specific C accumulation rates are calculated with IPCC default methods and values. C savings from bioenergy are given for a range of displacement factors (DFs), acknowledging the varying efficiency of bioenergy routes and technologies in fossil fuel displacement. A uniform spatial pattern is assumed for succession and bioenergy plantations, and the considered timeframes range from 20 to 100 years. For many parameter settings—in particular, longer timeframes and high DFs—bioenergy yields higher cumulative C savings than natural succession. Still, if woody biomass displaces liquid transport fuels or natural gas‐based electricity generation, natural succession is competitive or even superior for timeframes of 20–50 years. This finding has strong implications with climate and environmental policies: Freeing land for natural succession is a worthwhile low‐cost natural climate solution that has many co‐benefits for biodiversity and other ecosystem services. A considerable risk, however, is C stock losses (i.e., emissions) due to disturbances or land conversion at a later time.

## Graphical abstract

How can spare cropland be used more efficiently in terms of climate change mitigation? By growing short rotation coppice for bioenergy or allowing cropland to revert to its natural state. We show that the more beneficial option depends on the timeframe considered, biophysical conditions in the considered region, upstream emissions of fuel supply, and the efficiency of the particular bioenergy route in displacing fossil fuels.

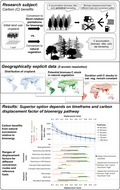

## 1 INTRODUCTION

On a global level, bioenergy currently holds the largest share of all renewable energy sources (REN21, 2018). Although its size is intensively discussed, the sustainable potential of biomass for energy is mostly considered to be substantial (e.g., Coelho et al., 2012; Creutzig et al., 2015; Dornburg et al., 2010; Haberl, Beringer, Bhattacharya, Erb, & Hoogwijk, 2010; Searle & Malins, 2015; WBGU, 2009), and global long‐term energy scenarios often show considerable increase in bioenergy production (Azar et al., 2010; Calvin et al., 2013; Creutzig et al., 2012; Daioglou, Doelman, Wicke, Faaij, & van Vuuren, 2019; Kitous et al., 2017; Krey, Luderer, Clarke, & Kriegler, 2014; Loftus, Cohen, Long, & Jenkins, 2015; OECD/IEA & IRENA, 2017; Riahi et al., 2017; Rogelj et al., 2016, 2018). It is generally assumed that large quantities of solid biomass could be sourced through intensified forest management and the conversion of unmanaged to managed forests (e.g., Fricko et al., 2017; Kraxner et al., 2013). Yet, concerns regarding the net carbon (C) impacts of increased forest harvests are rising. Due to the reduction in forest C stocks associated with increased use of forest biomass relative to a counterfactual scenario with lower harvests, it often takes considerable periods of time until forest bioenergy actually provides net C savings in comparison to fossil‐based reference systems (“fossil fuel parity time,” see Cherubini, Bright, & Strømman, 2012; Cintas et al., 2017; Gustavsson, Haus, Ortiz, Sathre, & Truong, 2015, 2016; Holtsmark, 2012; Hudiburg, Law, Wirth, & Luyssaert, 2011; Jonker, Junginger, & Faaij, 2014; Lamers & Junginger, 2013; McKechnie, Colombo, Chen, Mabee, & MacLean, 2011; Sterman, Siegel, & Rooney‐Varga, 2018; Vanhala, Repo, & Liski, 2013; Zanchi, Pena, & Bird, 2010, 2012). Depending on different influencing factors (management practices, tree species, types of fossil fuels being displaced, which parts of trees are used for energy and other uses, etc.), parity times vary from less than a year to several decades or even centuries (e.g., Agostini, Giuntoli, & Boulamanti, 2013; Bentsen, 2017; Buchholz, Hurteau, Gunn, & Saah, 2016; Mitchell, Harmon, & O'Connell, 2012).

An option for providing possibly large quantities of biomass without interfering with forest C stocks are purpose‐grown biomass plantations managed in short rotation (short rotation coppice; SRC). If established on current agricultural land, such plantations usually result in the buildup of C stocks in biota and soils rather than their reduction (e.g., Arevalo, Bhatti, Chang, & Sidders, 2011; Rytter, 2012; Verlinden et al., 2013) and usually provide higher energy yields per unit area and year than conventional energy crops like cereals or oilseeds (Boehmel, Lewandowski, & Claupein, 2008; Ericsson, Rosenqvist, & Nilsson, 2009; WBGU, 2009). Hence, provided that agricultural land can be diverted from food and feed production without impairing food security (Haberl et al., 2011), SRC appears as an attractive climate change mitigation measure. Scenario results of integrated assessment models also often show large‐scale deployment of energy plantations (e.g., Daioglou et al., 2019; Fricko et al., 2017; Kraxner et al., 2013).

However, land used for SRC could also be left to natural succession, that is, revert to natural ecosystems (usually natural forests), thereby acting as a potentially significant long‐term C sink. The world's croplands are mostly located in ecological zones that would revert to forests if left undisturbed, but there are also cropland areas located in regions characterized as shrublands, desert, or steppe (see Table S1). We therefore use the term “natural succession” (rather than “reforestation”), meaning any kind of regrowth of natural vegetation. Following the IPCC (Intergovernmental Panel on Climate Change) definition, we use the term “natural forest” for forest composed of indigenous trees as contrasted with plantations. We are aware that such forests would, for a long time, differ strongly from natural old‐growth forests without human use for centennial timeframes, which is the meaning usually attached to the notion of “natural forests” among conservation ecologists.

Contributions of bioenergy based on SRC (SRC‐based bioenergy) and natural succession to the reduction in atmospheric C are here referred to as “carbon benefits” (C benefits). This definition of C benefits differs from the C benefit index recently proposed by Searchinger, Wirsenius, Beringer, and Dumas (2018), which relates greenhouse gas emissions to a standardized land‐based product index allowing comparisons across products. In contrast, we here quantify reductions in atmospheric C resulting from two different land uses.

Depending on site‐specific conditions (climate, ecological zone), the considered timeframe, and the efficiency of the respective bioenergy pathways, natural succession might represent a worthwhile alternative to fossil fuel substitution with biomass from energy plantations. Erb et al. (2018) have recently highlighted the high relevance of natural vegetation as C storage and the massive effects of land use on C stocks, including land management without land‐cover conversion (e.g., forestry). Numerous studies have investigated C trade‐offs between forest management aiming at fossil fuel displacement and setting aside forests to maximize C sequestration (e.g., Cintas et al., 2017; Mitchell et al., 2012; Taeroe, Mustapha, Stupak, & Raulund‐Rasmussen, 2017; Vanhala et al., 2013), but few studies have compared C benefits of natural succession with those of SRC for energy. Albanito et al. (2016) have shown that for a timeframe of 20 years, reforestation of cropland would be superior to bioenergy from SRC in terms of their C balance on 17% of all global cropland areas. However, their assumptions regarding the average amount of fossil C being displaced per unit of biomass‐derived C are quite optimistic, as they assumed a high displacement factor (DF) of 0.878 (see below). In contrast, Marland and Schlamadinger (1997) assumed a default value of DF = 0.6 and a DF range of 0–1.0 in a sensitivity analysis. Assuming equal growth rates in afforestation and short rotation plantations, they found that for low DF and a timeframe of 40 years, afforestation achieves greater C benefits than bioenergy, whereas the situation is reversed for a 100‐year timeframe and/or high DF. Similarly, considering three specific bioenergy technologies, Baral and Guha (2004) found that it takes 30, 51, and 65 years, respectively, until biomass use for energy becomes superior to afforestation if initial biomass accumulation in afforestation and SRC are equal. Yet, for the southern United States, they assume biomass accumulation in afforestation to actually be significantly slower; in this case, bioenergy from SRC cultures would be generally superior to afforestation.

These studies illustrate that it depends on various factors whether natural succession (nSucc) or SRC‐based bioenergy (BE) provides higher cumulative C benefits per unit area. A systematic comparison of the respective C benefits has so far not been worked out.

The aim of this study is to fill this gap by investigating the subject on a global scale, taking into account regionally diverse influencing parameters. We systematically analyze the effect of largely arbitrary methodological decisions (such as the timeframe considered, the temporal pattern of land conversion, the assumed use of the biomass, and the fossil fuel‐based system displaced).

More specifically, this study aims at answering the following research questions: How do nSucc and BE compare in terms of cumulative C benefits, with regard to the predominating biophysical conditions in different world regions? To what extend does the superior option depend on the considered bioenergy pathway, and the assumptions on the fossil fuel and conversion route it displaces? What are the main influencing factors determining the superior option, and what do the findings imply for global climate mitigation strategies and policies?

C benefits denote total cumulative carbon savings resulting from either option per unit area over a defined period of time. C benefits from nSucc and BE are always compared for the same area and spatial distribution of land. Potential differences in albedo effects of natural vegetation and SRC are not considered. Moreover, we do not consider economic aspects of the two C mitigation options, implications for landowners or policy measures for incentivizing SRC or natural succession on agricultural land. Such issues are beyond the scope of this biophysical assessment of C benefits and must be addressed in subsequent research.

In this study, we only consider short rotation plantations (e.g., willow, poplar, eucalyptus) that are typically harvested in periods of 2–5 years. Conventional energy crops (e.g., oilseeds, cereals) as well as perennial grasses (e.g., switchgrass, giant reed) are not within the scope of this work. We calculate C stock changes in accordance with IPCC methods and largely based on IPCC default data (IPCC, 2006b). This approach comes at the expense of simplified growth dynamics (constant annual C accumulation, i.e., linear growth), but implies a highest possible degree of agreement with established data and methods. It is intended as a first generic approach to an underresearched issue of land‐based climate change mitigation. Assessment of potential options at the project level will require more detailed site‐specific approaches.

We further check the robustness of our results with sensitivity analyses regarding uncertain parameter values and by considering nonlinear growth functions (logistic functions or, where available, Chapman‐Richards growth curves; see Pienaar & Turnbull, 1973). With regard to bioenergy technologies, we consider conversion routes for heat, electricity, and liquid transport fuel production and also investigate the relevance of C emissions in upstream processes (i.e., biomass harvest and transport as well as fossil fuel supply chains). Bioenergy with carbon capture and storage (see Fridahl & Lehtveer, 2018; Fuss et al., 2014) is not considered.

## 2 MATERIALS AND METHODS

**Figure 1:**
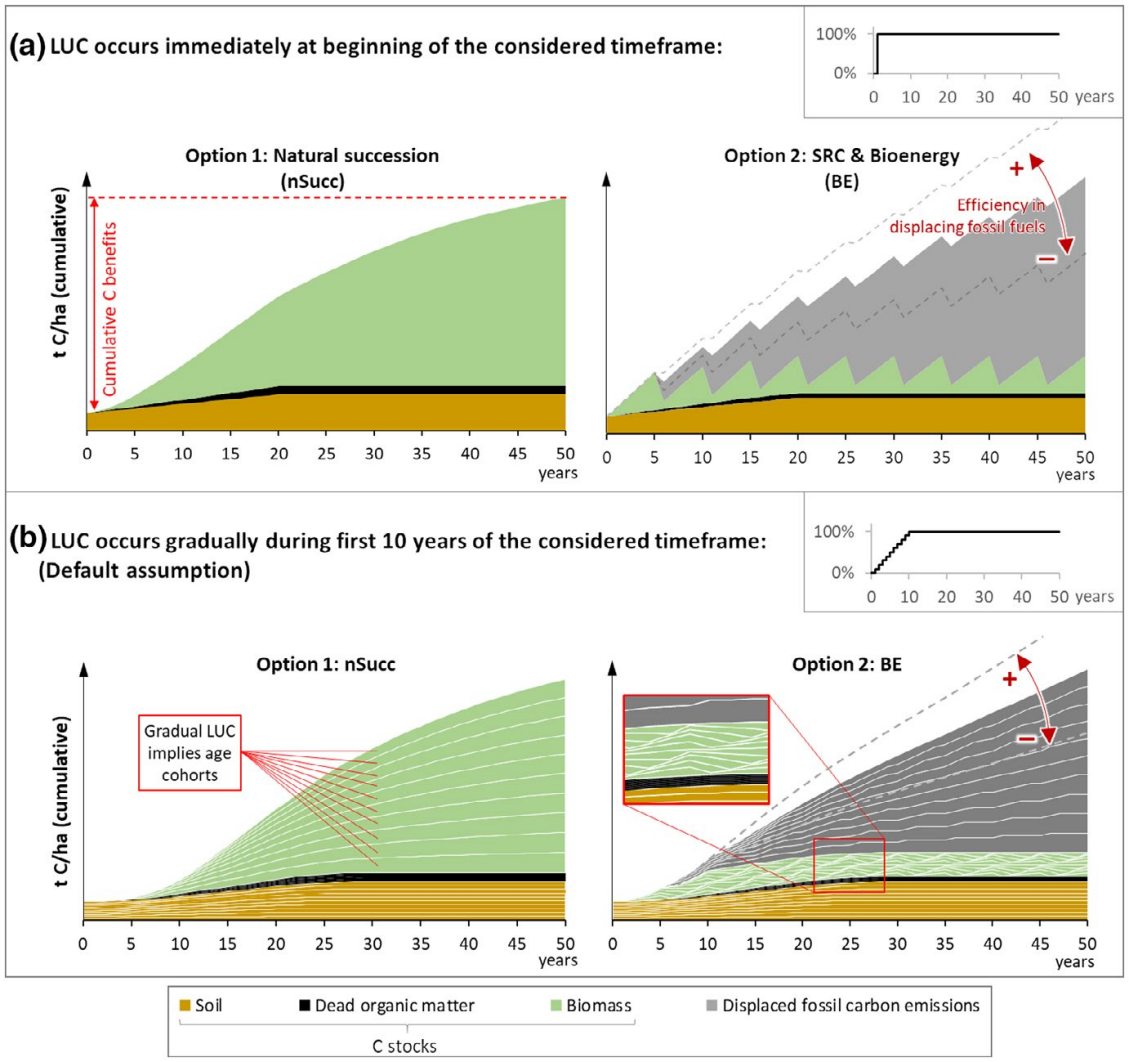
Schematic illustration of the research topic: (a) is based on the assumption that 100% of the considered area is converted from cropland to natural succession (left) or short rotation coppice (right) immediately at the beginning of the considered timeframe, whereas in (b), a transition period of 10 years is assumed. This results in constant biomass C stocks in short rotation coppice (SRC) plantations as compared to the “sawtooth profile” shown in (a). The situation shown in (b) represents our default case

The following figure illustrates the fundamentals of the research topic and the methodological approach: The left panel of Figure 1a shows an exemplary development of C stocks if one hectare of cropland is left to nSucc. Following IPCC methods, we differentiate between the C pools biomass (including above‐ and belowground biomass; BM_above and BM_below), dead organic matter (DOM; comprising litter and dead wood), and soil (soil organic carbon; SOC). If we assume that this land‐use change (LUC) occurs after a final crop harvest, the initial C stock in year zero is essentially made up of SOC. The development in subsequent years is characterized by relatively slow growth during the first years, followed by accelerating and later on subsiding C accumulation. According to Tier 1 assumptions of IPCC (2006b), soil and DOM C stocks can be assumed to reach a new equilibrium state within 20 years since LUC. The cumulative C benefits of nSucc (CB_nSucc_) correspond to the total difference in C stocks between the initial and the final year of the considered timeframe:

(1) $${\mathrm{CB}}_{\mathrm{nSucc}}=\Delta C_{\mathrm{nSucc}}^{\mathrm{BM}\_\mathrm{above}}+\Delta C_{\mathrm{nSucc}}^{\mathrm{BM}\_\mathrm{below}}+\Delta C_{\mathrm{nSucc}}^{\mathrm{DOM}}+\Delta C^{\mathrm{SOC}}$$

In contrast, the development of C stocks in SRC (right panel of Figure 1a) is characterized by rapid initial growth, followed by a depletion of the aboveground biomass C stock after each rotation period. The harvested biomass is used to substitute fossil fuels. Hence, fossil C emissions are avoided, contributing to the C benefits of this option. The amount of displaced C emissions depends on the efficiency of the considered bioenergy pathway and the specifics of the fossil‐based counterpart. We generally assume that energy from biomass—on the level of final energy, that is, electricity, district heat, or transport fuel—displaces the same amount of final energy originating from a fossil fuel‐based conversion chain. The facts that the “displacement ratio” might actually be smaller than 1 (e.g., due to rebound effects) and that bioenergy might displace other renewable energy technologies are disregarded (see York, 2012).

The figure also illustrates the effects of varying DFs: If biomass displaces a high‐carbon fossil fuel (e.g., coal), using a high‐efficiency bioenergy technology, more fossil C is displaced per unit of biogenic C (upper dashed line in the right panel of Figure 1a) than in case of rather inefficient bioenergy plants and displacement of low‐carbon fossil fuels (e.g., natural gas; lower dashed line). In case of SRC‐based bioenergy, the bulk of C benefits apparently originate from fossil fuel displacement rather than C stock changes.

Figure 1a represents a situation where a certain area is converted at the beginning of the considered timeframe. Especially in the context of global deployment of SRC systems, it is more realistic to assume that this process extends over a period of several years. This is reflected in Figure 1b, with an assumed “transition period” of 10 years. Hence, one‐tenth of the considered area is assumed to be converted annually in the first 10 years of the timeframe. Contrary to the previous case, total C stocks are now composed of 10 age cohorts for each C stock type. These age cohorts are shown as separate areas in Figure 1b.

For SRC, the assumption of gradual LUC implies annual harvests and constant biomass C stocks after the transition period, because biomass removal from a patch that was ready for harvest is compensated by biomass growth on immature patches (see close‐up detail in right panel). Hence, assuming gradual LUC is not only more realistic than immediate LUC but also has the advantage that it leads to continuous C benefit curves instead of “sawtooth profiles.” It therefore constitutes a default assumption in our modeling approach. The default transition period is 10 years, and an alternative duration of 20 years is assumed in a sensitivity analysis.

In the case of BE, C benefits (CB_BE_) result from C stock changes and displacement of fossil fuels (CB_fuel_disp_):

(2) $$\begin{array}{c} {\mathrm{CB}}_{\mathrm{BE}}=\Delta C_{\mathrm{BE}}^{\mathrm{BM}\_\mathrm{above}}+\Delta C_{\mathrm{BE}}^{\mathrm{BM}\_\mathrm{below}}+\Delta C_{\mathrm{BE}}^{\mathrm{DOM}}\\ \;\;+\Delta C^{\mathrm{SOC}}+{\mathrm{CB}}_{\mathrm{fuel}\_\mathrm{disp}} \end{array}$$

CB_fuel_disp_ is calculated according to the following equation, with DF ranging from 0.1 to 1:

(3) $${\mathrm{CB}}_{\mathrm{fuel}\_\mathrm{disp}}=Y\left(1-L_{\mathrm{supply}\_\mathrm{chain}}\right)\left(\mathrm{TF}-\frac{\mathrm{TP}}{2}\right)\mathrm{DF}$$

where* Y* denotes average biomass yields per year (measured in tons of C), *L*_supply_chain_ is relative biomass loss along the biomass supply chain, TF is the considered timeframe, and TP is the assumed transition period of LUC.

Apart from a global perspective, we differentiate between 11 world regions (adopted from Haberl et al., 2011) and consider timeframes of 20, 30, 40, 50, 70, and 100 years. We calculate C benefits on a per hectare basis, assuming that the considered area (where SRC plantations are established or nSucc takes place) is evenly distributed among all cropland areas of the considered region. By taking into account the respective cropland distribution among climate zones, soil types, and ecosystem zones, we obtain unique C accumulation curves for each world region. The underlying data are described in the following section.

### 2.1 Data

Whenever possible, we use default values provided for Tier 1 approaches in IPCC (2006b) for modeling C stock changes. These default data depend on ecological zones (net biomass growth in natural forests; Table 4.12), climate zones (default litter stocks in forests, Table 2.2), and soil types (SOC depends on soil types as well as climate; see Table 2.3). Belowground biomass stocks are derived from default root‐to‐shoot ratios according to Table 4.4.

Realistic forest growth curves are characterized by relatively low net C accumulation in the first few years since conversion (see Humpenöder et al., 2014; Winrock International, 2014). Based on data on tropical forest systems available from Winrock International (2014), we assume that during the first 3 years of natural succession, net biomass growth in all types of natural forests is one‐third of the respective IPCC default value. Thereafter, growth is assumed to continue with constant annual C uptake until the maximum C stock level of the respective ecosystem (Table 2.2 in IPCC, 2006b) is reached.

To investigate the relevance of the growth functions' shapes on our results, we alternatively assume S‐shaped growth functions. For tropical forests, we use growth functions according to Winrock International (2014), and for other ecological zones, logistic growth functions derived by curve fitting (see Supporting Information for details). Following IPCC Tier 1 methods, initial litter C stocks on cropland are assumed to be zero, and litter as well as soil C stock changes are assumed to occur linearly over a transition period of 20 years.

For natural vegetation in temperate steppe, we assume maximum above‐ and belowground biomass C stocks of 7 tons of dry mass per hectare (t_dry_/ha) and for deserts 2 t_dry_/ha (WBGU, 1998). Data on aboveground biomass C stocks in SRC are derived from yield estimates, biomass losses, and litter decay rates. From a thorough literature review (see Supporting Information), we concluded that data on potential net primary production (“NPP_pot_”) among world regions (Haberl et al., 2007) are good estimates for SRC yields when on‐site aboveground losses *L*_on‐site_ (due to herbivory, leaf shedding, etc.; see Clark et al., 2001) of 20% are assumed.

(4) $$Y={\mathrm{NPP}}_{\mathrm{pot}}\left(1-L_{\mathrm{on}-\mathrm{site}}\right)$$

Aboveground losses remaining on‐site turn into litter, providing the basis for our estimates of litter C stocks in SRC. In a sensitivity analysis, actual yields are varied by assuming a plus/minus 20% variation in NPP_pot_ and a range of 10%–30% for aboveground losses. Further losses along the wood chip supply chain are assumed to be 10% (based on Baral & Guha, 2004; Lenz, Idler, Hartung, & Pecenka, 2015; Wästerlund, Nilsson, & Gref, 2017). Belowground biomass in SRC is assumed 30% of aboveground biomass. Although data in the literature show considerable ranges of root‐to‐shoot ratios (Berhongaray, Verlinden, Broeckx, Janssens, & Ceulemans, 2017; Das & Chaturvedi, 2005; Heilman, Ekuan, & Fogle, 1994; Oliveira et al., 2018; Saugier, Roy, & Mooney, 2001), this ratio appears as a reasonable estimate. Dead wood is generally disregarded in IPCC Tier 1 methods and thus also not taken into account here. Literature data on SRC yields and details regarding litter C stock calculations in SRC plantations are provided in the Supporting Information.

**Figure 2:**
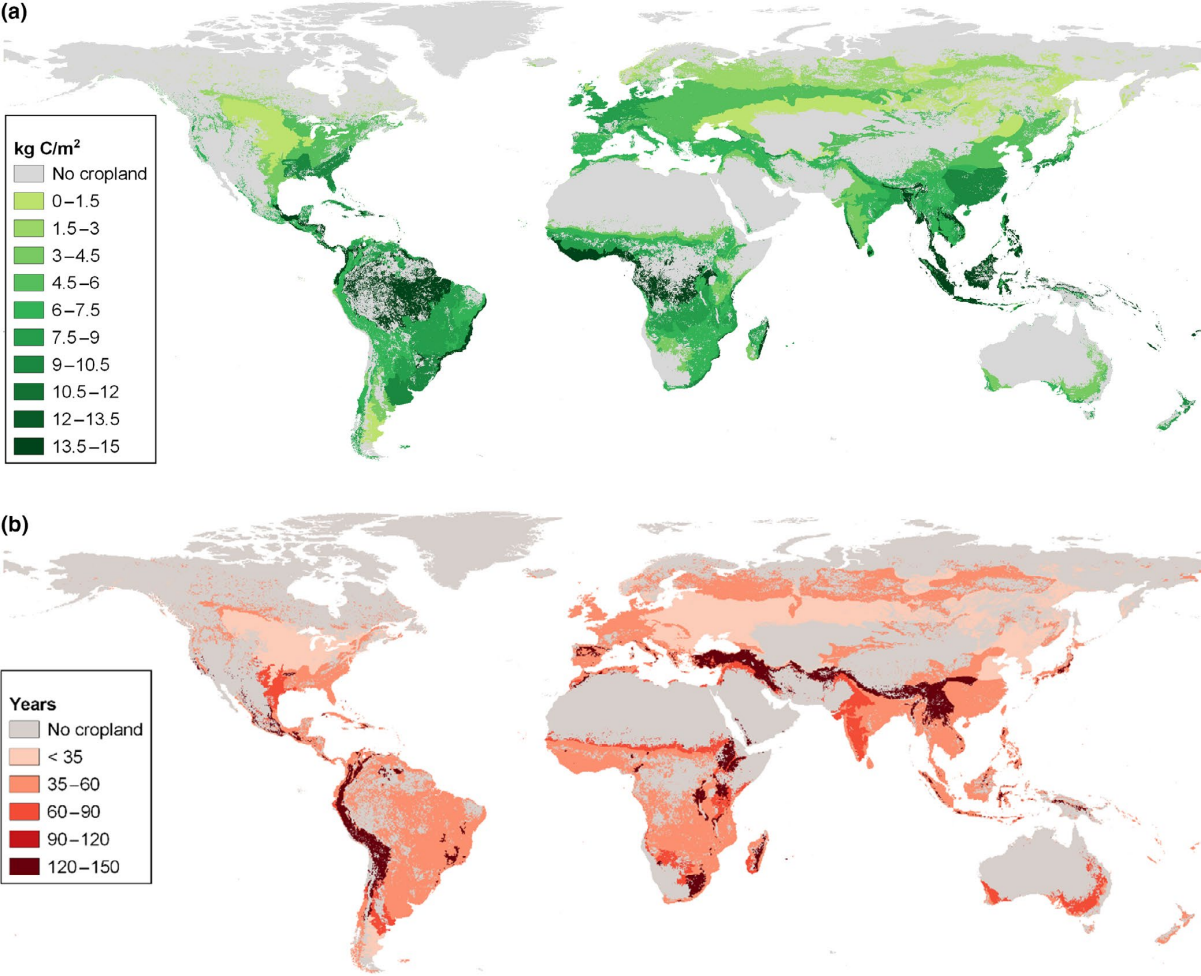
Illustration of natural vegetation growth patterns on grid cells with cropland. (a) Potential (maximum) aboveground biomass carbon stock. (b) Duration until maximum carbon stock is reached (own calculations and illustration based on IPCC, 2006b and data obtained from Erb et al., 2007; FAO, 2012; JRC, 2018)

We consider heterogenic biophysical conditions in the different world regions by determining the spatial distribution of total cropland areas in each region. We use global raster data on climate zones, ecological zones, and soil types with a resolution of 5 arc‐minutes. These data have been obtained from JRC (2018) (soil and climate), FAO (2012) (ecological zones), and Erb et al. (2007) (cropland distribution). To illustrate the regional differences in growth patterns of natural forests, Figure 2 shows world maps of potential aboveground biomass C stocks (i.e., the stable maximum when left undisturbed according to IPCC Tier 1 data; Figure 2a) and the time it takes until the maximum stock is reached (Figure 2b). Grid cells without cropland are shown in gray colour; thus, the maps also provide insight into the global distribution of croplands.

According to IPCC Guidelines, SOC stocks on cropland depend on soil types, climate, crop types (annual/perennial crops; paddy rice), and agricultural practices (tillage, input of residues, manure, etc.). Under Tier 1 approaches (see equation 2.25 in IPCC, 2006b), SOC stocks are determined by climate‐ and soil‐specific reference values given in tons of C per ha (table 2.3) and “relative stock change factors” that reflect the effects of the other influencing parameters (table 5.5). For natural forest as well as for plantations, stock change factors are generally equal to 1 under Tier 1, so there is generally no difference in SOC stocks between nSucc and BE. Nevertheless, to account for the entire C stock change in our model, we estimate C stocks at the beginning and the end of the considered timeframe. For initial SOC stocks (i.e., to be able to determine stock change factors for cropland from Table 5.5), we simplistically assume that all cropland has been continuously used for annual crops with medium input level. Different regional tillage practices are considered based on data by Prestele, Hirsch, Davin, Seneviratne, and Verburg (2018) (see Table S3).

### 2.2 Calculation of carbon DFs

To establish the link between the above‐described C benefit calculations, where DFs are arbitrary parameters being varied from 0.1 to 1, with actual bioenergy pathways, we calculate representative ranges of DFs for heat, electricity, and transport fuel production from wood chips. We consider state‐of‐the‐art technologies and efficiencies as well as expected efficiency improvements due to technological progress or implementation of novel conversion technologies, such as biomass integrated gasification combined cycle plants for power generation.

DF is defined as the ratio of fossil carbon emissions to biomass carbon emission for an equal amount of (final) energy supplied. Following Marland and Schlamadinger (1997), we calculate DF from the combustion emissions of wood chips (CE_BM_) and a reference fossil fuel (CE_fossil_), and the conversion efficiencies *η* of the considered bioenergy pathways (index “BE”) and the respective fossil‐based counterpart (index “fossil”):

(5) $${\mathrm{DF}}_{\mathrm{BE}}=\frac{\eta_{\mathrm{BE}}}{\eta_{\mathrm{fossil}}}\times \frac{{\mathrm{CE}}_{\mathrm{fossil}}}{{\mathrm{CE}}_{\mathrm{BM}}}$$

Depending on the bioenergy pathway, efficiencies represent thermal or electrical plant efficiencies or feedstock‐to‐fuel conversion efficiencies (of second‐generation biofuel production processes or petroleum refineries). Hence, DFs reflect (a) energy losses during the respective conversion from primary resource to final energy and (b) the different emission factors of biomass and fossil fuels.

In the case of combined heat and power (CHP) generation, we consider electricity as primary output and apply established allocation methods to determine the share of electricity‐related emissions. To consider upstream emissions of fuel supply (e.g., resulting from petroleum exploration, biomass harvesting and transport, etc.), we apply the following extended equation:

(6) $${\mathrm{DF}}_{\mathrm{BE}}^{\mathrm{incl}.\mathrm{UE}}=\frac{\eta_{\mathrm{BE}}}{\eta_{\mathrm{fossil}}}\times \frac{{\mathrm{CE}}_{\mathrm{fossil}}+{\mathrm{UE}}_{\mathrm{fossil}}}{{\mathrm{CE}}_{\mathrm{BM}}}-\frac{{\mathrm{UE}}_{\mathrm{BM}}}{{\mathrm{CE}}_{\mathrm{BM}}}$$

UE_BM_ and UE_fossil_ denote upstream emissions per energy unit of biomass and fossil fuel, respectively. Combustion emission factors are taken from IPCC Guidelines (IPCC, 2006a), and upstream emissions are based on literature data (Bradbury, Obeiter, Draucker, Wang, & Stevens, 2013; Brandt, 2011; EC, 2015; Giuntoli, Agostini, Edwards, & Marelli, 2015; Schweier et al., 2017; Scull et al., 2017). Depending on transport distances and modes, characteristics of fossil fuel deposits and other influencing factors, upstream emissions vary widely. For biomass as well as fossil fuels, we consider “best‐case” and “worst‐case” situations and investigate scenarios with the largest possible influence on DFs: “favorable” bioenergy scenarios, where biomass from a best‐case situation regarding upstream emissions is used to displace fossil fuel from a worst‐case situation, and “unfavorable” bioenergy scenarios, where the opposite is assumed.

Detailed information on the assumed technologies, allocation methods, combustion emission factors, and assumed ranges of upstream emissions are provided in the Supporting Information.

## 3 RESULTS

The results are presented in the following ways: First, we compare the relative performance of nSucc in relation to BE, with DF being varied from 0.1 to 1. The ratio (CB_nSucc_/CB_BE_) is referred to as “relative C benefits from nSucc.” Second, we present sensitivity analyses for uncertainties related to SRC, assuming transition periods of 10 (default) as well as 20 years. Third, we compare C benefits of nSucc and BE in absolute numbers for 11 world regions and elaborate on similarities and differences between the regions as well as the global weighted average. And fourth, we consider dynamic DFs and present scenarios for the timeframe 2020 to 2100. In the latter two cases, we take into account linear (default) as well as nonlinear growth functions for nSucc.

Table 1 gives an overview of the analyses and underlying parameter settings. The results are presented in the following four subsections.

**Table 1:** Overview of the analyses and underlying parameter settings

	Short description of analysis	Spatial disaggregation of presented results	Considered duration(s) of transition period	Considered type(s) of growth curves (nSucc)^a^	Considered displacement factor values^b^
1	Relative carbon benefits from nSucc	• Global only	• 10 years	• Linear	• 0.1 to 1
2	Sensitivity analysis (Monte Carlo sim.) regarding SRC yields and on‐site losses	• Global only	• 10 years • 20 years	• Linear	• 0.2, 0.5, 0.8 • 0.1 to 1 (Figure S8)
3	Absolute carbon benefits from nSucc and BE	• 11 world regions^c^ • Global	• 10 years	• Linear • Nonlinear	• 0.1 to 1
4	Scenarios with dynamic displacement factors	• Global only	• 10 years	• Linear • Nonlinear	• 2 scenarios (dynamic values)

a See Figure S2 in the Supporting Information.

b Displacement factors are constant during considered timeframe if not stated otherwise.

c See Figure S1 in the Supporting information.

### 3.1 Relative carbon benefits from natural succession

**Figure 3:**
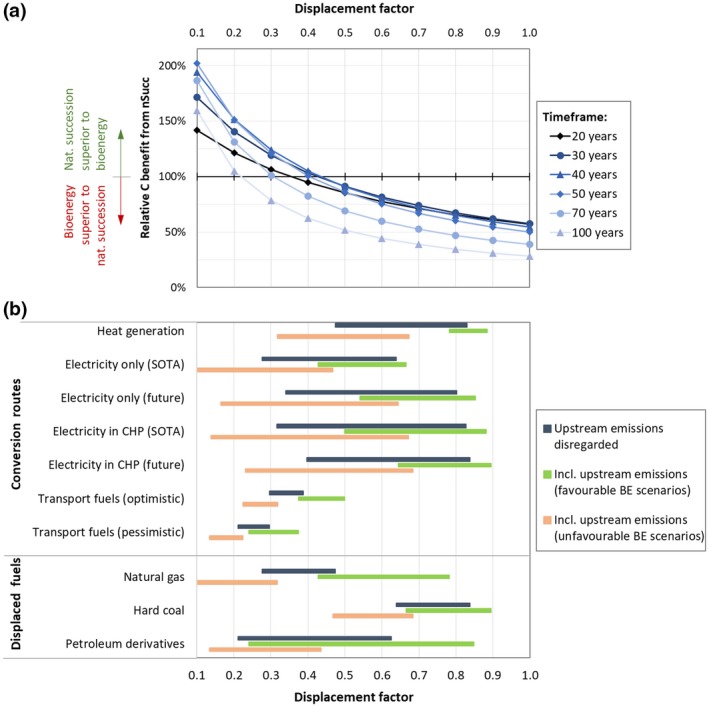
Relative C benefits of natural succession on global scale in the context of different bioenergy options: (a) the cumulative C benefits of natural succession relative to the C benefits from short rotation coppice (SRC)‐based bioenergy assuming different displacement factors and timeframes; (b) the displacement factors resulting from different biomass conversion routes and reference fossil fuels. Upstream emissions of biomass and fossil fuel supply chains are disregarded (dark gray bars) or reflect either favorable (green bars) or unfavorable bioenergy cases (apricot‐colored bars); see Supporting Information for underlying technology data, emission factors, upstream emissions and displacement factors for each technology pair

Figure 3a shows the cumulated C benefits from nSucc relative to those from BE under default parameter settings. Ratios above 100% mean that nSucc is superior to BE under the respective conditions, whereas ratios below 100% indicate superiority of BE. Each line represents results for a specific timeframe, with DF being varied from 0.1 to 1.

Results indicate that BE is superior to nSucc for many, but not all parameter settings. For intermediate DF values and timeframes, savings from nSucc are between 10% and 30% lower than those from BE. At a very low DF (0.2 or lower), C benefits from nSucc generally exceed those from BE, regardless of the timeframe, and at 0.3, it takes more than 70 years for BE to achieve higher cumulative C savings. In contrast, DF values at least slightly higher than 0.4 result in BE being superior to nSucc for all considered timeframes. Still, it is remarkable that even at high DF (i.e., 0.7 to 1.0), the carbon savings from nSucc are in the range of about 50%–75% of the savings from BE for timeframes up to 50 years.

The reason why relative C benefits from nSucc are surprisingly low for 20‐ and 30‐year timeframes and low DF is higher initial biomass growth in SRC than in nSucc. For low DF values and short timeframes, the C benefits from BE are largely due to C stock increase in SRC, whereas for medium to long timeframes (and generally for high DF values), C benefits from BE mostly originate from fossil fuel displacement.

Figure 3b associates the range of DF with bioenergy conversion routes and types of fossil fuels. If supply chain emissions of biomass as well as fossil fuels are disregarded, the DF of the considered bioenergy options range from about 0.2 to 0.85. The highest values are achieved when coal is displaced in heat, electricity, or CHP generation. Displacing natural gas results in DF in the range of 0.28–0.47, with heat‐only production being most effective. The large range for petroleum derivatives is due to relatively high values in heat‐only generation (0.63) and very low values in case of transport fuel displacement (0.21–0.39). This wide range for transport fuels originates from uncertainties regarding future conversion efficiencies of second‐generation (2G) biofuel plants that are considered by differentiating between “optimistic” and “pessimistic” projections in the literature.

The figure also illustrates the potentially large impact of supply chain emissions. “Unfavorable” bioenergy scenarios, characterized by long‐distance supply chains for biomass displacing efficient fossil fuel supply, exhibit DF ranges that are significantly lower than under exclusion of upstream emissions. The impact of high supply chain emissions is especially pronounced in case of biomass conversion routes with low efficiencies, such as second‐generation biofuel production or electricity generation without heat utilization. The overall range for unfavorable bioenergy scenarios is 0.1–0.68.

In contrast, if biomass is produced locally and displaces fossil fuels that are extracted with high energy input (such as unconventional sources of natural gas or petroleum from oil sands), the DF range for heat and electricity generation is 0.42–0.9. The DF of 2G biofuel production remains low at 0.22–0.37 for pessimistic projections and increases to up to 0.5 for the most optimistic projections.

### 3.2 Sensitivity analysis regarding SRC yields and on‐site losses

A sensitivity analysis for assessing uncertainties related to the productivity of SRC is implemented as Monte Carlo simulation (see Mooney, 1997; Morgan, Henrion, & Small, 1990) with 10^4^ runs. Random variables are NPP_pot_ (uniform distribution in the range from minus to plus 20% of the default values; see Supporting Information) and on‐site aboveground biomass losses *L*_on‐site_ (uniform distribution from 10% to 30% of NPP_pot_). Supply chain losses are kept constant at 10% of harvested biomass. The aggregate effect of the assumed ranges is that the highest possible value for energy plantation yields in each region is almost twice as high as the lowest possible value (see Figure S7).

**Figure 4:**
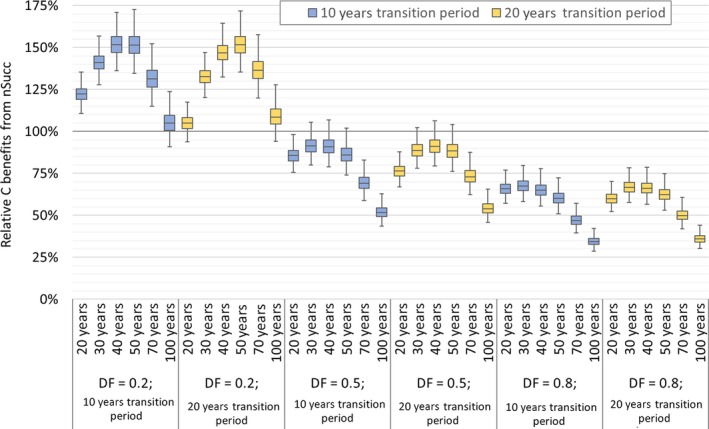
Sensitivity analysis regarding productivity of energy plantations (±20%) and on‐site biomass losses (±10%): Results from Monte Carlo simulations for exemplary displacement factors of 0.2, 0.5, and 0.8. (results for DF values from 0.2 to 1 are provided in the Supporting Information)

Figure 4 shows selected results, namely those for DF = 0.2, 0.5, and 0.8, as box plots with whiskers from minimum to maximum. Considering that the yield ranges are quite large, the interquartile ranges are surprisingly narrow. It is therefore concluded that the results (in terms of relative C benefits from nSucc) are quite robust to uncertainties related to energy plantation yields. However, this analysis does not account for possible bias in global average yield expectations, as samples for NPP_pot_ have been drawn for each world region individually.

Figure 4 also shows only minor differences for results based on a transition period of 20 years instead of the default 10 years. Only for timeframes of 20 years, there are noteworthy differences between the two cases, with longer transition periods having a detrimental effect on the performance of nSucc. Contrarily, for longer timeframes, a prolonged transition period results in improved performance of nSucc because the effect of biomass accumulation going into saturation—the reason for low relative C benefits from nSucc at long timeframes—is mitigated.

### 3.3 Absolute carbon benefits from SRC‐based bioenergy and natural succession for 11 world regions

**Figure 5:**
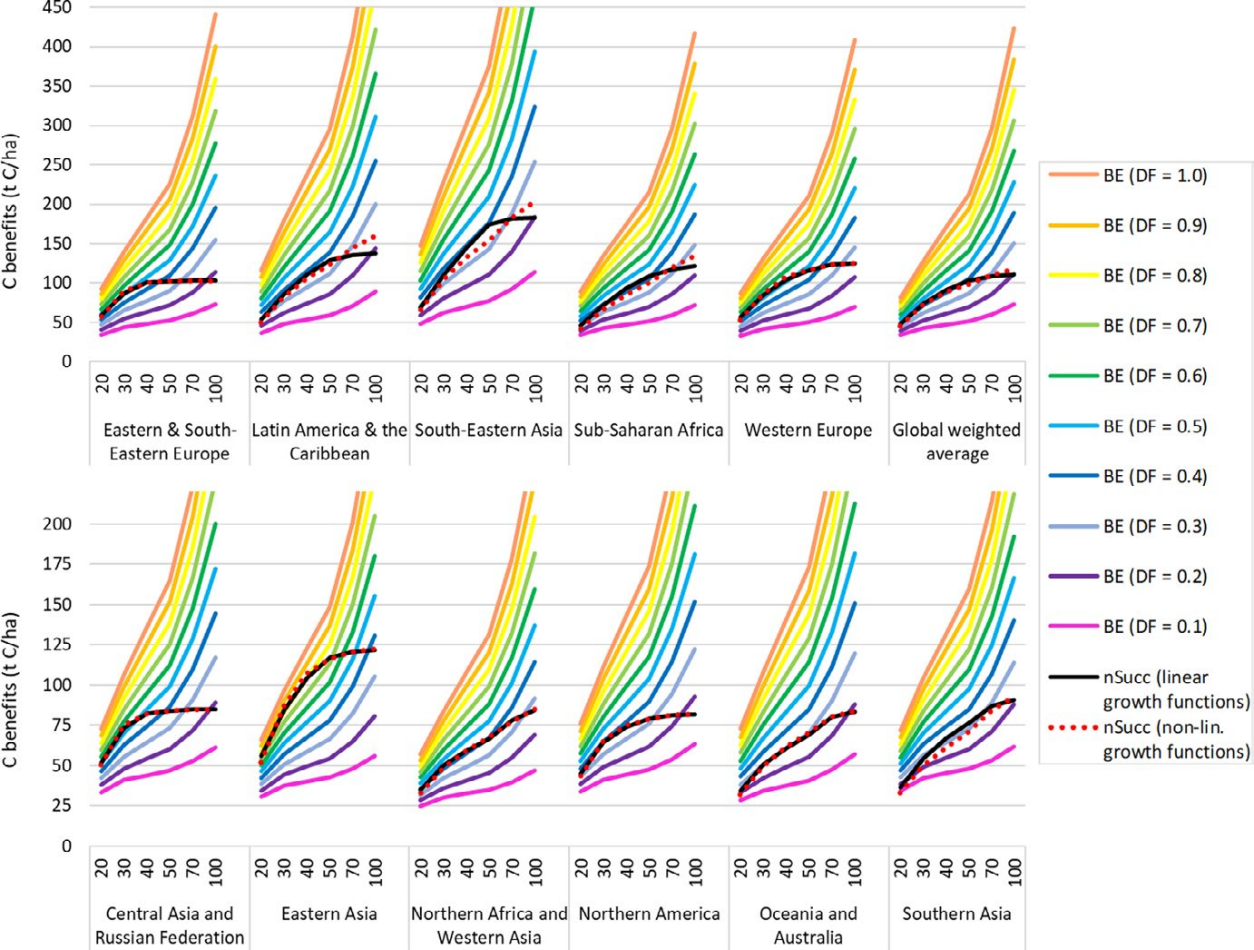
Cumulative carbon benefits from short rotation coppice‐based bioenergy (BE) and natural succession (nSucc) for 11 world regions and global weighted averages

Figure 5 illustrates that the absolute amounts of C benefits per hectare, resulting from nSucc as well as BE, vary considerably among world regions. Unsurprisingly, the highest values are achieved in tropical regions, namely South‐Eastern Asia and Latin America & the Caribbean. With regard to the performance of nSucc relative to BE (i.e., the position of the nSucc curves within the range covered by BE‐curves based on different DF), the results for most regions are quite similar to those for the weighted global average: For timeframes of 30–70 years, the C benefits from nSucc are in the range of BE‐curves with DF = 0.3–0.5. For a 100‐year timeframe, nSucc in most regions yields similar C benefits as BE with DF = 0.2. BE with DF = 0.6 typically shows somewhat higher C benefits for timeframes of up to 50 years; for even higher DF and timeframes, BE is clearly superior to nSucc everywhere.

Notwithstanding the quite large variation for a 20‐year timeframe,^1^ 1We consider the results for the timeframe of 20 years to be least reliable because they are strongly influenced by the uncertain rate of C accumulation in nSucc during the first few years. Our assumptions regarding this aspect are simplistic, and therefore, we refrain from an interpretation of the variation among world regions. the only truly conspicuous exception is Eastern Asia, where SRC yields are possibly underestimated. However, literature data for these world regions are sparse and considered too inconclusive for justifying an upwards correction. Still, we conclude that despite highly heterogeneous biophysical conditions among world regions, the performance of nSucc relative to BE is not entirely different.

Figure 5 also shows the C benefits resulting from alternative, nonlinear growth curves (see Figure S2). In the assumed Chapman‐Richards–based curves for tropical forest systems, the transition from high C accumulation rates toward saturation is very smooth and the saturation value higher than IPCC Tier 1 default values. This is obvious in the results for Latin America & the Caribbean, Sub‐Saharan Africa, and especially South‐Eastern Asia. For other ecological zones, we fitted logistic functions to the Tier‐1–based default linear growth curves. Regarding the results for nontropical world regions, it is obvious that substituting logistic growth curves for linear ones has very little effect on the results. Overall, the choice of growth function has some effect, but does not alter the general picture.

### 3.4 Scenarios with dynamic DFs

Considering the vast differences in DF depending on which conversion route is applied and which fossil fuel is displaced, estimating average DFs is challenging; for the present situation and even more for the future. However, we here present tentative assumptions regarding average values for the near future and possible developments until 2100. Being aware that such assumptions are highly speculative, the aim here is to emphasize the policy relevance of our analysis by putting it into the context of climate policy aims and timeframes.

We compare the cumulative C benefit curves for nSucc with those from two BE‐curves based on an optimistic (“HIGH”) and a pessimistic scenario (“LOW”) regarding fossil fuel displacement from 2020 to 2100. Building upon the ranges shown in Figure 3b, they are based on the following considerations: In the HIGH scenario, SRC‐based biomass is assumed to initially displace mainly coal and oil in heat and CHP generation, leading to an average DF of 0.7. In the LOW scenario, we assume that the displacement of natural gas is equally common, resulting in DF = 0.55. Assuming that global energy supply will be decarbonized in the second half of the 21st century (see UNFCCC, 2015), it is clear that “low‐hanging fruits” for mitigating carbon emissions will be harvested first. In other words, it is reasonable to assume that average DFs will decrease quite rapidly in earlier decades, as high‐carbon fossil fuels are gradually phased out. In the HIGH scenario, we therefore assume a decrease to 0.55 until 2050, based on the assumption that coal‐based energy production is becoming increasingly irrelevant. Until 2100, we assume a further reduction to 0.4, representing a mix of efficient 2G biofuel production (i.e., optimistic projections for technology development) and substitution of natural gas. The development in the LOW scenario (0.35 in 2050 and 0.25 in 2100) is conceivable under more ambitious decarbonization pathways with renewable energy dominating energy supply in 2050 and SRC‐based biomass being mainly used for 2G biofuel production in less efficient processes than in the HIGH scenario.

**Figure 6:**
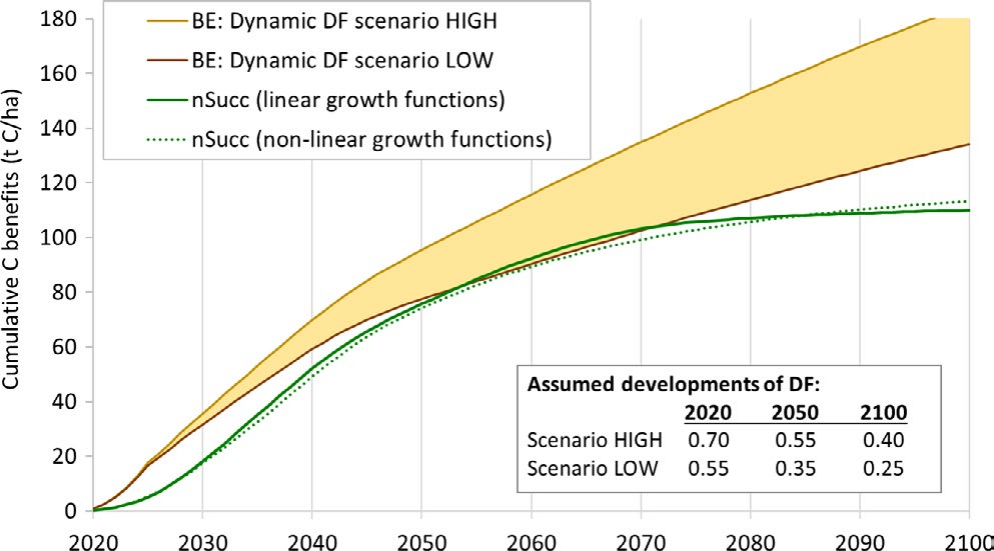
Cumulative carbon benefits from short rotation coppice‐based bioenergy (BE; based on two scenarios regarding the development of average displacement factors) and natural succession (nSucc; based on linear and nonlinear growth functions). DF values are given in the box in the lower right corner; intermediate values are interpolated linearly

Figure 6 shows the resulting developments of cumulative C benefits for BE and those for nSucc based on linear and nonlinear growth functions. This analysis reveals surprisingly little difference between the C benefits from nSucc and the scenario LOW, especially in the middle of the considered period. BE has significantly higher C benefits in the first decade due to faster C accumulation in SRC and relatively high DF during 2020–2030. But as a result of declining DF as well as accelerating C accumulation in natural vegetation, during 2050–2070, nSucc shows almost the same cumulative C benefits as BE in the scenario LOW. In the scenario HIGH, the C benefits from BE are about 25%–35% higher than in the other case of nSucc and scenario LOW. After 2070, C accumulation in nSucc declines and BE is again becoming increasingly superior, also in the scenario LOW. The decline in nSucc is slightly less pronounced if nonlinear growth functions are assumed, but still, the BE scenarios lead to clearly higher cumulative C benefits in the year 2100. Nevertheless, these results show that the C mitigation performance of nSucc in relation to SRC‐based bioenergy is generally remarkably high if we consider prospective shifts in fuel mixes and predominant biomass conversion routes.

## 4 DISCUSSION

Recent studies have argued that protection and enhancement of natural carbon sinks should seriously be considered as alternative to bioenergy (DeCicco & Schlesinger, 2018). Moreover, Griscom et al. (2017) have revealed the vast potentials of “natural climate solutions” (i.e., “better stewardship of land”) in mitigating climate change. We here contribute to this debate by comparing the C sink strength of natural succession on agricultural land in a systematic way and with global scope.

For low to medium DFs and timeframes of 20–50 years, natural succession is competitive or even superior to SRC‐based bioenergy in terms of C benefits per unit area. Why is this the case? C accumulation rates in energy plantations and natural vegetation both vary significantly throughout ecological zones, but are of a similar magnitude on individual sites. C stocks in natural vegetation increase steadily until they reach a (quasi‐) constant level after a period of time that ranges from few decades to over one century according to IPCC default values. In case of SRC, fossil fuel displacement must compensate for periodical depletion of aboveground biomass C stocks. However, the amount of (fossil‐based) C emissions displaced per unit of biogenic C used for energy is normally smaller than 1 due to usually lower conversion efficiencies and a relatively high emission factor of wood. If biomass displaces natural gas (for heat, electricity, or CHP) or oil derivatives via 2G biofuels, the DF is even smaller than 0.5. The lower the DF, the slower the process of compensating for C stock depletion in energy plantations.

The fact that C stocks in energy plantations are restored within relatively short periods after harvesting might foster the perception of “carbon neutrality,” and that net C savings are always achieved on short term, regardless of the efficiency of bioenergy use. This misperception stems from neglecting the counterfactual scenario, that is, C sequestration in natural vegetation; in fact, high efficiency in displacing fossil C—that is, high DFs—is of crucial importance.

We here applied IPCC Tier 1 approaches for modeling forest C accumulation that have the advantage of being relatively simple and building upon well‐agreed data (IPCC, 2006b). Their weaknesses, however, include simplified growth dynamics and exclusion of disturbances as well as deadwood stocks, which might actually be considerable (see Marchetti, 2005).^2^ 2No default values on deadwood are provided in IPCC (2006b) because literature data are not considered as statistically representative. However, previous guidelines (IPCC, 2003) suggest that average dead‐to‐live biomass ratios range from 0.11 to 0.2 for tropical, evergreen, and deciduous forests. Substituting linear growth functions for nonlinear ones did not significantly alter the results. Still, future research should be dedicated to this aspect, not only to have nSucc represented with realistic growth functions but also due to strong evidence that biomass C stocks in old‐growth forests are actually underestimated in current IPCC default data (see Keith, Mackey, & Lindenmayer, 2009; Luyssaert et al., 2008).

Further aspects that are disregarded here but deserve consideration include perennial energy grasses. We here considered only SRC, although energy grasses might be preferable in terms of biomass yields in some regions (see Albanito et al., 2016; Boehmel et al., 2008; Ericsson et al., 2009). We also neglected possible differences in albedo effects and soil C stocks between natural forests and SRC (following IPCC Tier 1 methods, SOC stocks are here considered to be equal) and natural disturbances. With regard to forest fires, Mitchell et al. (2012) argue that wildfires usually have a limited and temporary impact on C stocks because they mostly affect leaf litter and fine wood debris (see Campbell, Harmon, & Mitchell, 2012; Mitchell, Harmon, & O'Connell, 2009). Still, high‐intensity forest fires or severe storms may lead to massive C losses, cancelling out the C accumulation of many years or decades (see Hurteau & Brooks, 2011). Another considerable risk to climate mitigation through nSucc is intentional C stock depletion due to LUC at a later time. This must be avoided by adequate legislative measures (e.g., establishment of nature conservation areas) and monitoring, as long‐term permanence of C stocks is a core necessity of natural climate solutions.

With regard to bioenergy, there are environmental risks and dangers associated with large‐scale energy plantations (Beringer, Lucht, & Schaphoff, 2011; WBGU, 2009), whereas natural succession could yield considerable co‐benefits in terms of biodiversity and other ecosystem services (Griscom et al., 2017; Millennium Ecosystem Assessment, 2005). Furthermore, it is important to acknowledge that all carbon mitigation options that require agricultural land harbour the risk of driving food prices and aggravating hunger in less developed countries. Given global population growth and current trends in diets, it is questionable whether large areas of agricultural land can be freed up for bioenergy or natural succession without compromising food security (see Erb et al., 2016; Haberl et al., 2011).

Despite all uncertainties, our findings have strong implications for climate policies: Allowing agricultural land to follow a natural succession pathway must seriously be considered as an alternative climate change mitigation strategy to bioenergy. While we have analyzed which factors need to be considered when deciding which of these two options provides more climate benefits, we are aware that site‐based analyses using detailed and specific data are required for sound decisions at the level of individual projects. At the strategic level, however, our results suggest that natural succession has so far been greatly neglected. Considering that natural succession could provide considerable C savings (possibly of a similar magnitude as SRC‐based bioenergy) until the mid‐21st century at comparatively low costs (see Griscom et al., 2017; Kalt & Kranzl, 2011; WBGU, 2009), substituting this climate change mitigation option for SRC‐based bioenergy in decarbonization strategies could free up considerable financial resources for other renewable energy technologies such as wind or solar power, or investments in energy efficiency.

## References

[gcbb12626-bib-0001] Agostini, A. , Giuntoli, J. , & Boulamanti, A. (2013). Carbon accounting of forest bioenergy. Conclusions and recommendations from a critical literature review (JRC Technical reports). European Commission Joint Research Centre, Institute for Energy and Transport.

[gcbb12626-bib-0002] Albanito, F. , Beringer, T. , Corstanje, R. , Poulter, B. , Stephenson, A. , Zawadzka, J. , & Smith, P. (2016). Carbon implications of converting cropland to bioenergy crops or forest for climate mitigation: A global assessment. GCB Bioenergy, 8(1), 81–95. 10.1111/gcbb.12242

[gcbb12626-bib-0003] Arevalo, C. B. M. , Bhatti, J. S. , Chang, S. X. , & Sidders, D. (2011). Land use change effects on ecosystem carbon balance: From agricultural to hybrid poplar plantation. Agriculture, Ecosystems & Environment, 141(3–4), 342–349. 10.1016/j.agee.2011.03.013

[gcbb12626-bib-0004] Azar, C. , Lindgren, K. , Obersteiner, M. , Riahi, K. , van Vuuren, D. P. , den Elzen, K. M. G. J. , … Larson, E. D. (2010). The feasibility of low CO2 concentration targets and the role of bio‐energy with carbon capture and storage (BECCS). Climatic Change, 100(1), 195–202. 10.1007/s10584-010-9832-7

[gcbb12626-bib-0005] Baral, A. , & Guha, G. S. (2004). Trees for carbon sequestration or fossil fuel substitution: The issue of cost vs. carbon benefit. Biomass and Bioenergy, 27(1), 41–55. 10.1016/j.biombioe.2003.11.004

[gcbb12626-bib-0006] Bentsen, N. S. (2017). Carbon debt and payback time – Lost in the forest? Renewable and Sustainable Energy Reviews, 73, 1211–1217. 10.1016/j.rser.2017.02.004

[gcbb12626-bib-0007] Berhongaray, G. , Verlinden, M. S. , Broeckx, L. S. , Janssens, I. A. , & Ceulemans, R. (2017). Soil carbon and belowground carbon balance of a short‐rotation coppice: Assessments from three different approaches. GCB Bioenergy, 9(2), 299–313. 10.1111/gcbb.12369 28261329 PMC5310368

[gcbb12626-bib-0008] Beringer, T. , Lucht, W. , & Schaphoff, S. (2011). Bioenergy production potential of global biomass plantations under environmental and agricultural constraints. GCB Bioenergy, 3(4), 299–312. 10.1111/j.1757-1707.2010.01088.x

[gcbb12626-bib-0009] Boehmel, C. , Lewandowski, I. , & Claupein, W. (2008). Comparing annual and perennial energy cropping systems with different management intensities. Agricultural Systems, 96(1–3), 224–236. 10.1016/j.agsy.2007.08.004

[gcbb12626-bib-0010] Bradbury, J. , Obeiter, M. , Draucker, L. , Wang, W. , & Stevens, A. (2013). Clearing the air: Reducing upstream greenhouse gas emissions from U.S. natural gas systems (Working Paper) (p. 60). Washington, DC: World Resources Institute Retrieved from http://www.wri.org/publication/clearing-the-air

[gcbb12626-bib-0011] Brandt, A. R. (2011). Upstream greenhouse gas (GHG) emissions from Canadian oil sands as a feedstock for European refineries. Department of Energy Resources Engineering, Stanford University.

[gcbb12626-bib-0012] Buchholz, T. , Hurteau, M. D. , Gunn, J. , & Saah, D. (2016). A global meta‐analysis of forest bioenergy greenhouse gas emission accounting studies. GCB Bioenergy, 8(2), 281–289. 10.1111/gcbb.12245

[gcbb12626-bib-0013] Calvin, K. , Wise, M. , Klein, D. , McCollum, D. , Tavoni, M. , van der Zwaan, B. , & van Vuuren, D. P. (2013). A multi‐model analysis of the regional and sectoral roles of bioenergy in near‐and long‐term CO_2_ emissions reduction. Climate Change Economics, 4(04), 1340014 10.1142/s2010007813400149

[gcbb12626-bib-0014] Campbell, J. L. , Harmon, M. E. , & Mitchell, S. R. (2012). Can fuel‐reduction treatments really increase forest carbon storage in the western US by reducing future fire emissions? Frontiers in Ecology and the Environment, 10(2), 83–90. 10.1890/110057

[gcbb12626-bib-0015] Cherubini, F. , Bright, R. M. , & Strømman, A. H. (2012). Site‐specific global warming potentials of biogenic CO_2_ for bioenergy: Contributions from carbon fluxes and albedo dynamics. Environmental Research Letters, 7(4), 045902 10.1088/1748-9326/7/4/045902

[gcbb12626-bib-0016] Cintas, O. , Berndes, G. , Cowie, A. L. , Egnell, G. , Holmström, H. , Marland, G. , & Ågren, G. I. (2017). Carbon balances of bioenergy systems using biomass from forests managed with long rotations: Bridging the gap between stand and landscape assessments. GCB Bioenergy, 9(7), 1238–1251. 10.1111/gcbb.12425

[gcbb12626-bib-0017] Clark, D. A. , Brown, S. , Kicklighter, D. W. , Chambers, J. Q. , Thomlinson, J. R. , & Ni, J. (2001). Measuring net primary production in forests: Concepts and field methods. Ecological Applications, 11(2), 356–370. 10.1890/1051-0761(2001)011[0356:MNPPIF]2.0.CO;2

[gcbb12626-bib-0018] Coelho, S. T. , Agbenyega, O. , Agostini, A. , Erb, K.‐H. , Haberl, H. , Hoogwijk, M. , … Leemans, R. (2012 ). Land and water: Linkages to bioenergy In Johansson T. B., Nakicenovic N., Patwardhan A., & Gomez‐Echeverri L. (Eds.), Global Energy Assessment (GEA) (pp. 1459–1526). Cambridge: Cambridge University Press 10.1017/CBO9780511793677.026

[gcbb12626-bib-0019] Creutzig, F. , Popp, A. , Plevin, R. , Luderer, G. , Minx, J. , & Edenhofer, O. (2012). Reconciling top‐down and bottom‐up modelling on future bioenergy deployment. Nature Climate Change, 2(5), 320–327. 10.1038/nclimate1416

[gcbb12626-bib-0020] Creutzig, F. , Ravindranath, N. H. , Berndes, G. , Bolwig, S. , Bright, R. , Cherubini, F. , … Masera, O. (2015). Bioenergy and climate change mitigation: An assessment. GCB Bioenergy, 7(5), 916–944. 10.1111/gcbb.12205

[gcbb12626-bib-0021] Daioglou, V. , Doelman, J. C. , Wicke, B. , Faaij, A. , & van Vuuren, D. P. (2019). Integrated assessment of biomass supply and demand in climate change mitigation scenarios. Global Environmental Change, 54, 88–101. 10.1016/j.gloenvcha.2018.11.012

[gcbb12626-bib-0022] Das, D. K. , & Chaturvedi, O. P. (2005). Structure and function of *Populus deltoides* agroforestry systems in eastern India: 1. Dry matter dynamics. Agroforestry Systems, 65(3), 215–221. 10.1007/s10457-005-1266-2

[gcbb12626-bib-0023] DeCicco, J. M. , & Schlesinger, W. H. (2018). Reconsidering bioenergy given the urgency of climate protection. Proceedings of the National Academy of Sciences of the United States of America, 115(39), 9642–9645. 10.1073/pnas.1814120115 30254086 PMC6166821

[gcbb12626-bib-0024] Dornburg, V. , van Vuuren, D. , van de Ven, G. , Langeveld, H. , Meeusen, M. , Banse, M. , … Faaij, A. (2010). Bioenergy revisited: Key factors in global potentials of bioenergy. Energy & Environmental Science, 3(3), 258 10.1039/b922422j

[gcbb12626-bib-0025] EC . (2015). Study on actual GHG data for diesel, petrol, kerosene and natural gas. Final report. European Commission, DG ENER.

[gcbb12626-bib-0026] Erb, K.‐H. , Gaube, V. , Krausmann, F. , Plutzar, C. , Bondeau, A. , & Haberl, H. (2007). A comprehensive global 5 min resolution land‐use data set for the year 2000 consistent with national census data. Journal of Land Use Science, 2(3), 191–224. 10.1080/17474230701622981

[gcbb12626-bib-0027] Erb, K.‐H. , Kastner, T. , Plutzar, C. , Bais, A. L. S. , Carvalhais, N. , Fetzel, T. , … Luyssaert, S. (2018). Unexpectedly large impact of forest management and grazing on global vegetation biomass. Nature, 553, 73–76. 10.1038/nature25138 29258288 PMC5756473

[gcbb12626-bib-0028] Erb, K.‐H. , Lauk, C. , Kastner, T. , Mayer, A. , Theurl, M. C. , & Haberl, H. (2016). Exploring the biophysical option space for feeding the world without deforestation. Nature Communications, 7, 11382 10.1038/ncomms11382 PMC4838894 27092437

[gcbb12626-bib-0029] Ericsson, K. , Rosenqvist, H. , & Nilsson, L. J. (2009). Energy crop production costs in the EU. Biomass and Bioenergy, 33(11), 1577–1586. 10.1016/j.biombioe.2009.08.002

[gcbb12626-bib-0030] FAO . (2012). Global ecological zones for FAO forest reporting: 2010 Update (Forest Resources Assessment Working Paper 179). Rome: Food and Agriculture Organisation of the United Nations.

[gcbb12626-bib-0031] Fricko, O. , Havlik, P. , Rogelj, J. , Klimont, Z. , Gusti, M. , Johnson, N. , … Riahi, K. (2017). The marker quantification of the Shared Socioeconomic Pathway 2: A middle‐of‐the‐road scenario for the 21st century. Global Environmental Change, 42, 251–267. 10.1016/j.gloenvcha.2016.06.004

[gcbb12626-bib-0032] Fridahl, M. , & Lehtveer, M. (2018). Bioenergy with carbon capture and storage (BECCS): Global potential, investment preferences, and deployment barriers. Energy Research & Social Science, 42, 155–165. 10.1016/j.erss.2018.03.019

[gcbb12626-bib-0033] Fuss, S. , Canadell, J. G. , Peters, G. P. , Tavoni, M. , Andrew, R. M. , Ciais, P. , … Yamagata, Y. (2014). Betting on negative emissions. Nature Climate Change, 4, 850 10.1038/nclimate2392

[gcbb12626-bib-0034] Giuntoli, J. , Agostini, A. , Edwards, R. , & Marelli, L. (2015). Solid and gaseous bioenergy pathways: Input values and GHG emissions: Calculated according to the methodology set in COM(2010) 11 and SWD(2014) 259. Luxembourg: Publications Office Retrieved from http://bookshop.europa.eu/uri?target=EUB:NOTICE:LDNA27215:EN:HTML

[gcbb12626-bib-0035] Griscom, B. W. , Adams, J. , Ellis, P. W. , Houghton, R. A. , Lomax, G. , Miteva, D. A. , … Fargione, J. (2017). Natural climate solutions. Proceedings of the National Academy of Sciences of the United States of America, 114(44), 11645–11650. 10.1073/pnas.1710465114 29078344 PMC5676916

[gcbb12626-bib-0036] Gustavsson, L. , Haus, S. , Ortiz, C. A. , Sathre, R. , & Truong, N. L. (2015). Climate effects of bioenergy from forest residues in comparison to fossil energy. Applied Energy, 138, 36–50. 10.1016/j.apenergy.2014.10.013

[gcbb12626-bib-0037] Gustavsson, L. , Haus, S. , Ortiz, C. A. , Sathre, R. , & Truong, N. L. (2016). Corrigendum to “Climate effects of bioenergy from forest residues in comparison to fossil energy” [Appl. Energy 138 (2015) 36–50]. Applied Energy, 170, 490–493. 10.1016/j.apenergy.2016.02.087

[gcbb12626-bib-0038] Haberl, H. , Beringer, T. , Bhattacharya, S. C. , Erb, K.‐H. , & Hoogwijk, M. (2010). The global technical potential of bio‐energy in 2050 considering sustainability constraints. Current Opinion in Environmental Sustainability, 2(5–6), 394–403. 10.1016/j.cosust.2010.10.007 24069093 PMC3778854

[gcbb12626-bib-0039] Haberl, H. , Erb, K.‐H. , Krausmann, F. , Bondeau, A. , Lauk, C. , Müller, C. , … Steinberger, J. K. (2011). Global bioenergy potentials from agricultural land in 2050: Sensitivity to climate change, diets and yields. Biomass and Bioenergy, 35(12), 4753–4769. 10.1016/j.biombioe.2011.04.035 22211004 PMC3236288

[gcbb12626-bib-0040] Haberl, H. , Erb, K. H. , Krausmann, F. , Gaube, V. , Bondeau, A. , Plutzar, C. , … Fischer‐Kowalski, M. (2007). Quantifying and mapping the human appropriation of net primary production in earth's terrestrial ecosystems. Proceedings of the National Academy of Sciences of the United States of America, 104(31), 12942–12947. 10.1073/pnas.0704243104 17616580 PMC1911196

[gcbb12626-bib-0041] Heilman, P. , Ekuan, G. , & Fogle, D. (1994). Above‐and below‐ground biomass and fine roots of 4‐year‐old hybrids of *Populus trichocarpa* × *Populus deltoides* and parental species in short‐rotation culture. Canadian Journal of Forest Research, 24, 1186–1192. 10.1139/x94-156

[gcbb12626-bib-0042] Holtsmark, B. (2012). Harvesting in boreal forests and the biofuel carbon debt. Climatic Change, 112(2), 415–428. 10.1007/s10584-011-0222-6

[gcbb12626-bib-0043] Hudiburg, T. W. , Law, B. E. , Wirth, C. , & Luyssaert, S. (2011). Regional carbon dioxide implications of forest bioenergy production. Nature Climate Change, 1, 419–423. 10.1038/nclimate1264

[gcbb12626-bib-0044] Humpenöder, F. , Popp, A. , Dietrich, J. P. , Klein, D. , Lotze‐Campen, H. , Bonsch, M. , … Müller, C. (2014). Investigating afforestation and bioenergy CCS as climate change mitigation strategies. Environmental Research Letters, 9(6), 064029 10.1088/1748-9326/9/6/064029

[gcbb12626-bib-0045] Hurteau, M. D. , & Brooks, M. L. (2011). Short‐ and long‐term effects of fire on carbon in US dry temperate forest systems. BioScience, 61(2), 139–146. 10.1525/bio.2011.61.2.9

[gcbb12626-bib-0048] IPCC . (2003). Good practice guidance for land use, land‐use change and forestry. Kanagawa, Japan: IGES.

[gcbb12626-bib-0046] IPCC . (2006a). 2006 IPCC guidelines for national greenhouse gas inventories. Volume 2: Energy (Prepared by the National Greenhouse Gas Inventories Programme). Japan: IGES.

[gcbb12626-bib-0047] IPCC . (2006b). 2006 IPCC guidelines for national greenhouse gas inventories. Volume 4: Agriculture, forestry and other land use (Prepared by the National Greenhouse Gas Inventories Programme). Japan: IGES.

[gcbb12626-bib-0049] Jonker, J. G. G. , Junginger, M. , & Faaij, A. (2014). Carbon payback period and carbon offset parity point of wood pellet production in the South‐eastern United States. GCB Bioenergy, 6(4), 371–389. 10.1111/gcbb.12056

[gcbb12626-bib-0050] JRC . (2018). Renewable energy directive. Thematic data layers for commission decision of [10 June 2010] on guidelines for the calculation of land carbon stocks for the purpose of Annex V to Directive 2009/28/EC. Joint Research Centre of the European Commission. Retrieved from https://esdac.jrc.ec.europa.eu/projects/renewable-energy-directive

[gcbb12626-bib-0051] Kalt, G. , & Kranzl, L. (2011). Assessing the economic efficiency of bioenergy technologies in climate mitigation and fossil fuel replacement in Austria using a techno‐economic approach. Applied Energy, 88(11), 3665–3684. 10.1016/j.apenergy.2011.03.014

[gcbb12626-bib-0052] Keith, H. , Mackey, B. G. , & Lindenmayer, D. B. (2009). Re‐evaluation of forest biomass carbon stocks and lessons from the world's most carbon‐dense forests. Proceedings of the National Academy of Sciences of the United States of America, 106(28), 11635–11640. 10.1073/pnas.0901970106 19553199 PMC2701447

[gcbb12626-bib-0053] Kitous, A. , Keramidas, K. , Vandyck, T. , Saveyn, B. , Van Dingenen, R. , Spadaro, J. V. , & Holland, M. (2017). Global energy and climate outlook 2017: How climate policies improve air quality. Global energy trends and ancillary benefits of the Paris Agreement. Luxembourg: Publications Office of the European Union 10.2760/474356

[gcbb12626-bib-0054] Kraxner, F. , Nordström, E.‐M. , Havlík, P. , Gusti, M. , Mosnier, A. , Frank, S. , … Obersteiner, M. (2013). Global bioenergy scenarios – Future forest development, land‐use implications, and trade‐offs. Biomass and Bioenergy, 57, 86–96. 10.1016/j.biombioe.2013.02.003

[gcbb12626-bib-0055] Krey, V. , Luderer, G. , Clarke, L. , & Kriegler, E. (2014). Getting from here to there – Energy technology transformation pathways in the EMF27 scenarios. Climatic Change, 123(3–4), 369–382. 10.1007/s10584-013-0947-5

[gcbb12626-bib-0056] Lamers, P. , & Junginger, M. (2013). The ‘debt’ is in the detail: A synthesis of recent temporal forest carbon analyses on woody biomass for energy. Biofuels, Bioproducts and Biorefining, 7(4), 373–385. 10.1002/bbb.1407

[gcbb12626-bib-0057] Lenz, H. , Idler, C. , Hartung, E. , & Pecenka, R. (2015). Open‐air storage of fine and coarse wood chips of poplar from short rotation coppice in covered piles. Biomass and Bioenergy, 83, 269–277. 10.1016/j.biombioe.2015.09.018

[gcbb12626-bib-0058] Loftus, P. J. , Cohen, A. M. , Long, J. C. S. , & Jenkins, J. D. (2015). A critical review of global decarbonization scenarios: What do they tell us about feasibility?: A critical review of global decarbonization scenarios. Wiley Interdisciplinary Reviews: Climate Change, 6(1), 93–112. 10.1002/wcc.324

[gcbb12626-bib-0059] Luyssaert, S. , Schulze, E.‐D. , Börner, A. , Knohl, A. , Hessenmöller, D. , Law, B. E. , … Grace, J. (2008). Old‐growth forests as global carbon sinks. Nature, 455(7210), 213–215. 10.1038/nature07276 18784722

[gcbb12626-bib-0060] Marchetti, M. (2005). Monitoring and indicators of forest biodiversity in Europe: From ideas to operationality. Joensuu: European Forest Institute Retrieved from http://lib.ugent.be/catalog/rug01:001041024

[gcbb12626-bib-0061] Marland, G. , & Schlamadinger, B. (1997). Forests for carbon sequestration or fossil fuel substitution? A sensitivity analysis. Biomass and Bioenergy, 13(6), 389–397. 10.1016/S0961-9534(97)00027-5

[gcbb12626-bib-0062] McKechnie, J. , Colombo, S. , Chen, J. , Mabee, W. , & MacLean, H. L. (2011). Forest bioenergy or forest carbon? Assessing trade‐offs in greenhouse gas mitigation with wood‐based fuels. Environmental Science & Technology, 45(2), 789–795. 10.1021/es1024004 21142063

[gcbb12626-bib-0063] Millennium Ecosystem Assessment (Program) . (Ed.). (2005). Ecosystems and human well‐being: Synthesis. Washington, DC: Island Press.

[gcbb12626-bib-0064] Mitchell, S. R. , Harmon, M. E. , & O'Connell, K. E. B. (2009). Forest fuel reduction alters fire severity and long‐term carbon storage in three Pacific Northwest ecosystems. Ecological Applications, 19(3), 643–655. 10.1890/08-0501.1 19425428

[gcbb12626-bib-0065] Mitchell, S. R. , Harmon, M. E. , & O'Connell, K. E. B. (2012). Carbon debt and carbon sequestration parity in forest bioenergy production. GCB Bioenergy, 4(6), 818–827. 10.1111/j.1757-1707.2012.01173.x

[gcbb12626-bib-0066] Mooney, C. (1997). Monte Carlo simulation. Thousand Oaks, CA: SAGE Publications 10.4135/9781412985116

[gcbb12626-bib-0067] Morgan, M. G. , Henrion, M. , & Small, M. (1990). Uncertainty: A guide to dealing with uncertainty in quantitative risk and policy analysis. Cambridge; New York: Cambridge University Press Retrieved from http://lib.ugent.be/catalog/rug01:000237360

[gcbb12626-bib-0068] OECD/IEA and IRENA . (2017). Perspectives for the energy transition – Investment needs for a low‐carbon energy system. Paris: IEA.

[gcbb12626-bib-0069] Oliveira, N. , Rodríguez‐Soalleiro, R. , Pérez‐Cruzado, C. , Cañellas, I. , Sixto, H. , & Ceulemans, R. (2018). Above‐ and below‐ground carbon accumulation and biomass allocation in poplar short rotation plantations under Mediterranean conditions. Forest Ecology and Management, 428, 57–65. 10.1016/j.foreco.2018.06.031

[gcbb12626-bib-0070] Pienaar, L. V. , & Turnbull, K. J. (1973). The Chapman‐Richards generalization of Von Bertalanffy's growth model for basal area growth and yield in even‐aged stands. Forest Science, 19(1), 2–22. 10.1093/forestscience/19.1.2

[gcbb12626-bib-0071] Prestele, R. , Hirsch, A. L. , Davin, E. L. , Seneviratne, S. I. , & Verburg, P. H. (2018). A spatially explicit representation of conservation agriculture for application in global change studies. Global Change Biology, 24(9), 4038–4053. 10.1111/gcb.14307 29749125 PMC6120452

[gcbb12626-bib-0072] REN21 . (2018). Renewables 2018. Global status report. Paris: REN21.

[gcbb12626-bib-0074] Riahi, K. , van Vuuren, D. P. , Kriegler, E. , Edmonds, J. , O'Neill, B. C. , Fujimori, S. , … Tavoni, M. (2017). The shared socioeconomic pathways and their energy, land use, and greenhouse gas emissions implications: An overview. Global Environmental Change, 42, 153–168. 10.1016/j.gloenvcha.2016.05.009

[gcbb12626-bib-0075] Rogelj, J. , den Elzen, M. , Höhne, N. , Fransen, T. , Fekete, H. , Winkler, H. , … Meinshausen, M. (2016). Paris agreement climate proposals need a boost to keep warming well below 2 °C. Nature, 534(7609), 631–639. 10.1038/nature18307 27357792

[gcbb12626-bib-0076] Rogelj, J. , Popp, A. , Calvin, K. V. , Luderer, G. , Emmerling, J. , Gernaat, D. , … Tavoni, M. (2018). Scenarios towards limiting global mean temperature increase below 1.5 °C. Nature Climate Change, 8(4), 325–332. 10.1038/s41558-018-0091-3

[gcbb12626-bib-0077] Rytter, R.‐M. (2012). The potential of willow and poplar plantations as carbon sinks in Sweden. Biomass and Bioenergy, 36, 86–95. 10.1016/j.biombioe.2011.10.012

[gcbb12626-bib-0078] Saugier, B. , Roy, J. , & Mooney, H. A. (2001). Terrestrial global productivity. London: Academic Press.

[gcbb12626-bib-0079] Schweier, J. , Molina‐Herrera, S. , Ghirardo, A. , Grote, R. , Díaz‐Pinés, E. , Kreuzwieser, J. , … Becker, G. (2017). Environmental impacts of bioenergy wood production from poplar short‐rotation coppice grown at a marginal agricultural site in Germany. GCB Bioenergy, 9(7), 1207–1221. 10.1111/gcbb.12423

[gcbb12626-bib-0080] Scull, B. D. , Kaddoura, S. , Chen, K. , Gyourgis, N. , Liu, Y. , Miller, S. G. , … Yan, E. (2017). Upstream emissions of coal and gas. New York, NY; Columbia: University, School of International and Public Affairs.

[gcbb12626-bib-0081] Searchinger, T. D. , Wirsenius, S. , Beringer, T. , & Dumas, P. (2018). Assessing the efficiency of changes in land use for mitigating climate change. Nature, 564(7735), 249–253. 10.1038/s41586-018-0757-z 30542169

[gcbb12626-bib-0082] Searle, S. , & Malins, C. (2015). A reassessment of global bioenergy potential in 2050. GCB Bioenergy, 7(2), 328–336. 10.1111/gcbb.12141

[gcbb12626-bib-0083] Sterman, J. D. , Siegel, L. , & Rooney‐Varga, J. N. (2018). Does replacing coal with wood lower CO_2_ emissions? Dynamic lifecycle analysis of wood bioenergy. Environmental Research Letters, 13(1), 015007 10.1088/1748-9326/aaa512

[gcbb12626-bib-0084] Taeroe, A. , Mustapha, W. F. , Stupak, I. , & Raulund‐Rasmussen, K. (2017). Do forests best mitigate CO_2_ emissions to the atmosphere by setting them aside for maximization of carbon storage or by management for fossil fuel substitution? Journal of Environmental Management, 197, 117–129. 10.1016/j.jenvman.2017.03.051 28351817

[gcbb12626-bib-0085] UNFCCC . (2015). Adoptation of the Paris agreement (21st conference of the parties). Paris: United Nations.

[gcbb12626-bib-0086] Vanhala, P. , Repo, A. , & Liski, J. (2013). Forest bioenergy at the cost of carbon sequestration? Current Opinion in Environmental Sustainability, 5(1), 41–46. 10.1016/j.cosust.2012.10.015

[gcbb12626-bib-0087] Verlinden, M. S. , Broeckx, L. S. , Zona, D. , Berhongaray, G. , De Groote, T. , Camino Serrano, M. , … Ceulemans, R. (2013). Net ecosystem production and carbon balance of an SRC poplar plantation during its first rotation. Biomass and Bioenergy, 56, 412–422. 10.1016/j.biombioe.2013.05.033

[gcbb12626-bib-0088] Wästerlund, I. , Nilsson, P. , & Gref, R. (2017). Influence of storage on properties of wood chip material. Journal of Forest Science, 63(4), 182–191. 10.17221/46/2016-JFS

[gcbb12626-bib-0089] WBGU . (1998). The accounting of biological sinks and sources under the Kyoto protocol: A step forwards or backwards for Global Environmental Protection? Bremerhaven: German Advisory Council on Global Change (WBGU).

[gcbb12626-bib-0090] WBGU . (2009). Future bioenergy and sustainable land use. London: Earthscan London and Sterling, VA.

[gcbb12626-bib-0091] Winrock International . (2014). AFOLU carbon calculator. The afforestation/reforestation tool: Underlying data and methods (p. 24). US Aid.

[gcbb12626-bib-0092] York, R. (2012). Do alternative energy sources displace fossil fuels? Nature Climate Change, 2(6), 441–443. 10.1038/nclimate1451

[gcbb12626-bib-0093] Zanchi, G. , Pena, N. , & Bird, N. (2010). The upfront carbon debt of bioenergy. Joanneum Research. Retrieved from http://www.nobiomassburning.org/OLD/BAP/Climate_Change_files/Bioenergy_Joanneum_Research.pdf

[gcbb12626-bib-0094] Zanchi, G. , Pena, N. , & Bird, N. (2012). Is woody bioenergy carbon neutral? A comparative assessment of emissions from consumption of woody bioenergy and fossil fuel. GCB Bioenergy, 4(6), 761–772. 10.1111/j.1757-1707.2011.01149.x

