# Supplementary material for: Natural climate solutions versus bioenergy: Can carbon benefits of natural succession compete with bioenergy from short rotation coppice?
Source: Glob Change Biol Bioenergy. 2019 Jun 13;11(11):1283–97. doi: 10.1111/gcbb.12626 (PMC6852302; doi:10.1111/gcbb.12626)
Supplement: Supplementary file 1 [file GCBB-11-1283-s001.pdf]

Natural climate solutions vs. bioenergy: Can carbon benefits of natural succession compete with bioenergy from short rotation coppice?

# – Supporting information –

Gerald KALT[a,1], Andreas MAYER[a], Michaela C. THEURL[a], Christian LAUK[a], Karl-Heinz ERB[a], Helmut HABERL[a]

a) Institute of Social Ecology Vienna (SEC)
University of Natural Resources & Life Sciences, Vienna (BOKU)
1070 Vienna, Schottenfeldgasse 29, Austria

1) Corresponding author; gerald.kalt@boku.ac.at, +43 1 47654-73744

Content

1 Spatial data 1
1.1 World regions 1
1.2 Cropland distribution among ecological zones and climate zones 1
1.3 Tillage 2
2 Biomass growth curves for natural succession 3
2.1 Biomass growth curves in ecological zones 3
2.2 Carbon accumulation curves in world regions 4
3 Energy plantation yields 5
4 Calculation of litter stocks in SRC 8
5 Calculation of displacement factors 9
5.1 Technologies with a single energy output 9
5.2 Combined heat and power generation 9
6 Displacement factors and underlying data 10
6.1 Emission factors and upstream emissions 10
6.2 Technology data 11
6.3 Displacement factors 14
7 Full results to the sensitivity analysis regarding yields and losses 15
Appendix: Glossary to section 5 16
Literature 17

1 Spatial data

1.1 World regions

The regional grouping (Fig. S1) has been adopted from previous studies (e.g. Erb et al., 2016; Haberl et al., 2011) and is based on the classification of the macro-geographical (continental) regions and geographical sub-regions as defined by the United Nations Statistical Division.

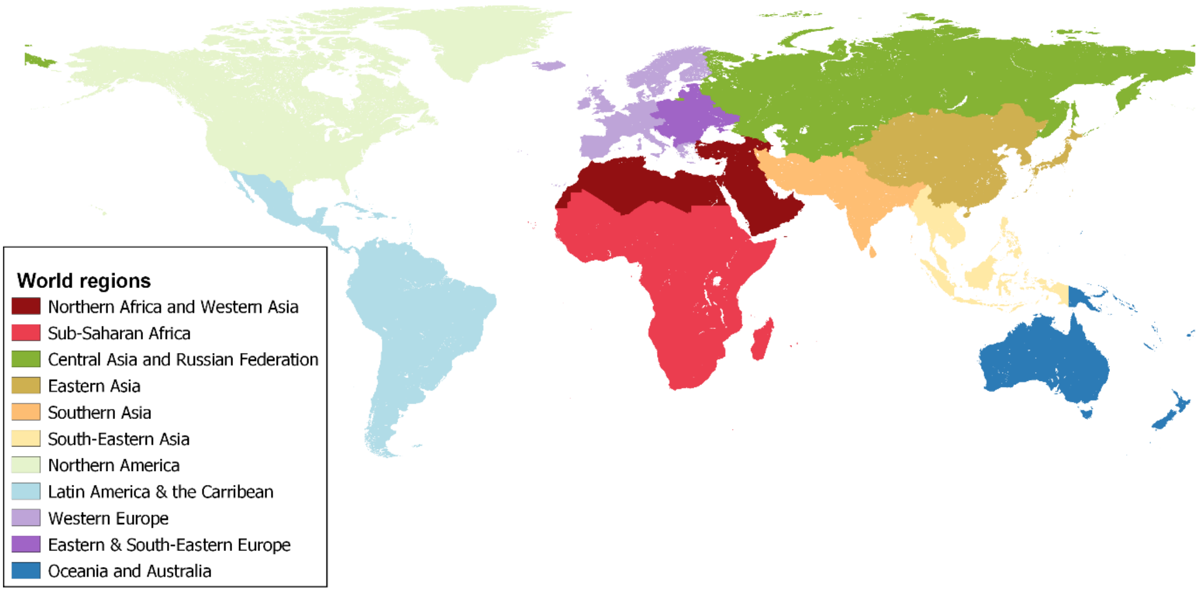

Figure S1. Regional grouping (own illustration based on Haberl et al., 2011)

1.2 Cropland distribution among ecological zones and climate zones

The following table S1 shows the distribution of cropland in each world region across ecological zones, as derived from the respective GIS data (cf. section 2.2 in the main article). These distributions, together with annual biomass growth data (Table 4.12 in IPCC 2006b), determine the rate of C accumulation in nSucc.

Table S2 shows the distribution of cropland among climate zones, which– according to the Tier 1 approach – determines the litter accumulation rate and stock (Table 2.2 in IPCC, 2006b).

Table S1. Distribution of cropland across ecological zones (own calculations)

	Northern Africa and Western Asia	Sub-Saharan Africa	Central Asia and Russian Federation	Eastern Asia	Southern Asia	South-Eastern Asia	Northern America	Latin America & the Carribean	Western Europe	Eastern & South-Eastern Europe	Oceania and Australia
Tropical rainforest	0%	17%	0%	0%	3%	61%	0%	20%	0%	0%	1%
Tropical moist forest	0%	24%	0%	1%	13%	13%	0%	32%	0%	0%	0%
Tropical dry forest	0%	20%	0%	0%	24%	20%	0%	12%	0%	0%	0%
Tropical shrubland	0%	19%	0%	0%	36%	0%	0%	0%	0%	0%	3%
Tropical desert	6%	1%	0%	0%	8%	0%	0%	0%	0%	0%	0%
Tropical mountain system	2%	12%	0%	0%	1%	6%	0%	10%	0%	0%	1%
Subtropical humid forest	1%	1%	0%	42%	1%	0%	14%	13%	0%	0%	3%
Subtropical dry forest	28%	1%	0%	0%	0%	0%	1%	1%	32%	2%	13%
Subtropical steppe	26%	0%	0%	0%	3%	0%	9%	5%	0%	0%	66%
Subtropical desert	4%	0%	0%	0%	0%	0%	0%	2%	0%	0%	2%
Subtropical mountain system	25%	3%	0%	7%	10%	0%	0%	3%	7%	0%	0%
Temperate oceanic forest	0%	0%	0%	0%	0%	0%	0%	0%	47%	2%	7%
Temperate continental forest	5%	0%	39%	22%	0%	0%	22%	0%	4%	76%	0%
Temperate steppe	0%	0%	26%	10%	1%	0%	44%	2%	0%	17%	0%
Temperate desert	1%	0%	7%	2%	0%	0%	2%	0%	0%	0%	0%
Temperate mountain system	0%	0%	6%	15%	0%	0%	4%	0%	5%	4%	5%
Boreal coniferous forest	0%	0%	16%	0%	0%	0%	5%	0%	5%	0%	0%
Boreal tundra woodland	0%	0%	0%	0%	0%	0%	0%	0%	0%	0%	0%
Boreal mountain system	0%	0%	6%	0%	0%	0%	0%	0%	1%	0%	0%

Table S2. Distribution of cropland across climate zones (own calculations)

	Northern Africa and Western Asia	Sub-Saharan Africa	Central Asia and Russian Federation	Eastern Asia	Southern Asia	South-Eastern Asia	Northern America	Latin America & the Carribean	Western Europe	Eastern & South-Eastern Europe	Oceania and Australia
Warm Temperate moist	3%	3%	0%	25%	2%	1%	22%	5%	17%	2%	5%
Warm Temperate dry		7%	10%	24%	8%	0%	11%	15%	38%	22%	70%
Cool Temperate moist	4%	0%	33%	8%	1%	0%	26%	1%	39%	42%	2%
Cool Temperate dry	7%	0%	43%	25%	2%	0%	30%	3%	5%	33%	0%
Boreal moist	0%	0%	10%	1%	0%	0%	0%	0%	0%	0%	0%
Boreal dry	0%	0%	4%	1%	0%	0%	0%	0%	0%	0%	0%
Tropical montane	2%	22%	0%	1%	1%	5%	0%	6%	0%	0%	1%
Tropical Wet	0%	4%	0%	0%	5%	55%	0%	12%	0%	0%	1%
Tropical moist	0%	27%	0%	15%	32%	36%	7%	43%	0%	0%	0%
Tropical dry	27%	37%	0%	0%	51%	3%	4%	16%	0%	0%	21%

1.3 Tillage

According to IPCC methods (IPCC, 2006b), SOC stocks on cropland are influenced by tillage practices. To consider this in our calculations of SOC stock changes, we used literature data tillage (shares of "conservation agriculture") per country to derive estimates of tillage shares in the different world regions. The following table, based on Prestele et al. (2018), summarizes the assumed tillage shares.

Table S3. Assumed shares of full, reduced and zero tillage for estimating SOC stock changes

Region	Zero tillage	Reduced tillage	Full tillage
Northern Africa and Western Asia	0.2%	0.0%	99.8%
Sub-Saharan Africa	0.7%	0.2%	99.1%
Central Asia and Russian Federation	4.0%	1.0%	95.0%
Eastern Asia	5.9%	1.5%	92.7%
Southern Asia	0.8%	0.2%	99.0%
South-Eastern Asia	0.0%	0.0%	100.0%
Northern America	27.3%	9.2%	63.5%
Latin America & the Caribbean	33.9%	5.5%	60.6%
Western Europe	3.4%	19.8%	76.8%
Eastern & South-Eastern Europe	2.3%	5.9%	91.8%
Oceania and Australia	37.1%	9.3%	53.6%

Source: based on Prestele et al. (2018)

2 Biomass growth curves for natural succession

The following sections illustrate the assumed biomass growth curves assumed for natural succession.

2.1 Biomass growth curves in ecological zones

Figure S2 illustrates the assumed aboveground biomass growth curves for ecological zones. Growth curves for steppe and deserts are not shown; the total of above- and belowground biomass C stocks in these ecological zones is assumed 7 and 2 t_{dry}/ha, respectively (WBGU, 1998).

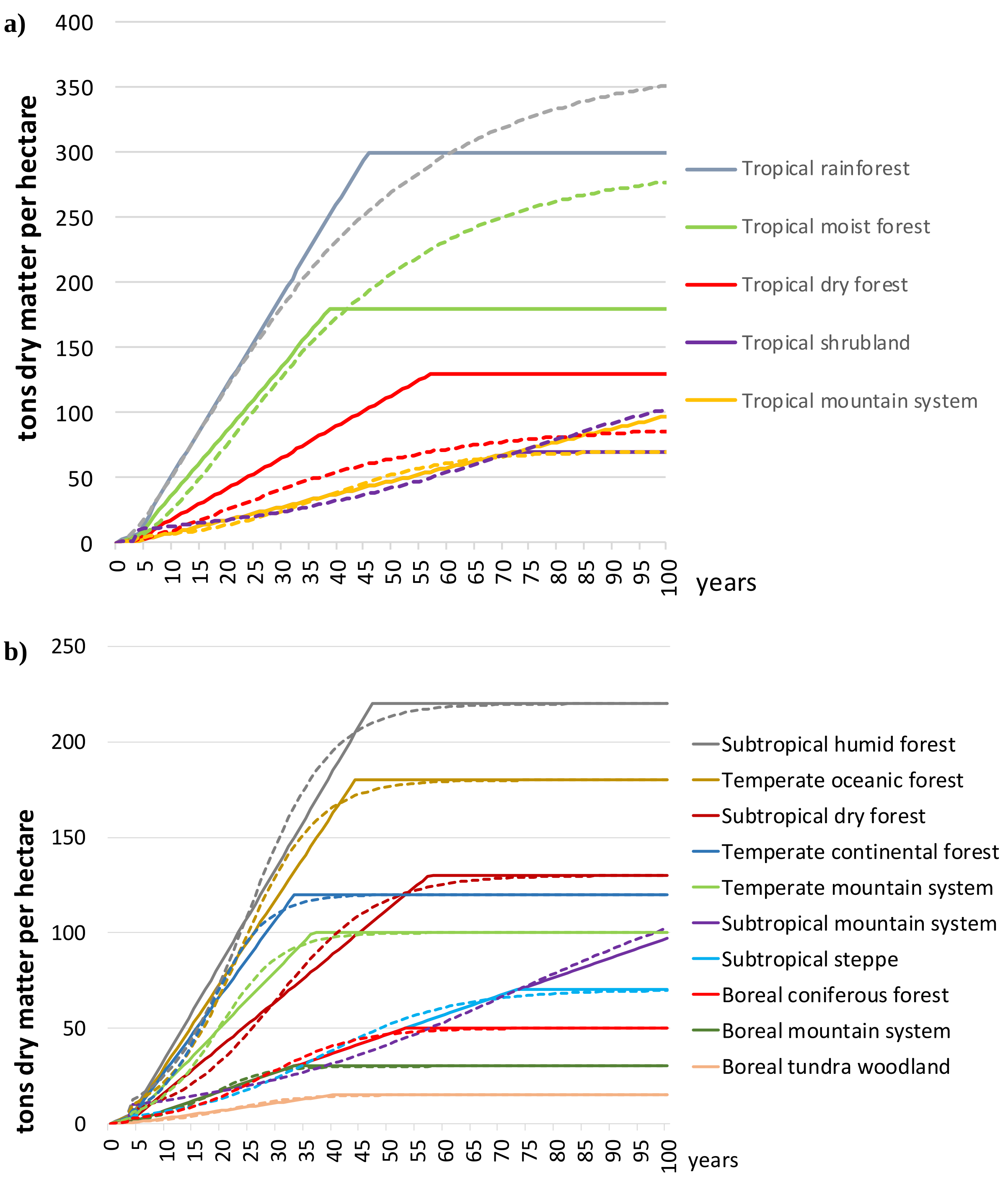

Figure S2. Aboveground biomass accumulation curves for ecosystem zones. Solid lines represent linear growth curves (our default curves) based on IPCC Tier 1 default data and dashed lines non-linear growth functions based on Winrock International (2014) (tropical forest systems) and logistic curve fitting (others), respectively.
Source: own calculations based on default data from IPCC (2006b) and Winrock International (2014).

2.2 Carbon accumulation curves in world regions

Figure S3 illustrates the C accumulation curves resulting from the data shown in Figure S2, Table S1 and default root-to-shoot ratios according to IPCC (2006b) Tier 1. The curves shown here are based on linear growth curves. In contrast to Figure S2, transition periods are considered here; solid lines are based on an assumed 10 years transition period (default

assumption), dashed lines on a 20 years transition period (alternative assumption in the sensitivity analysis).

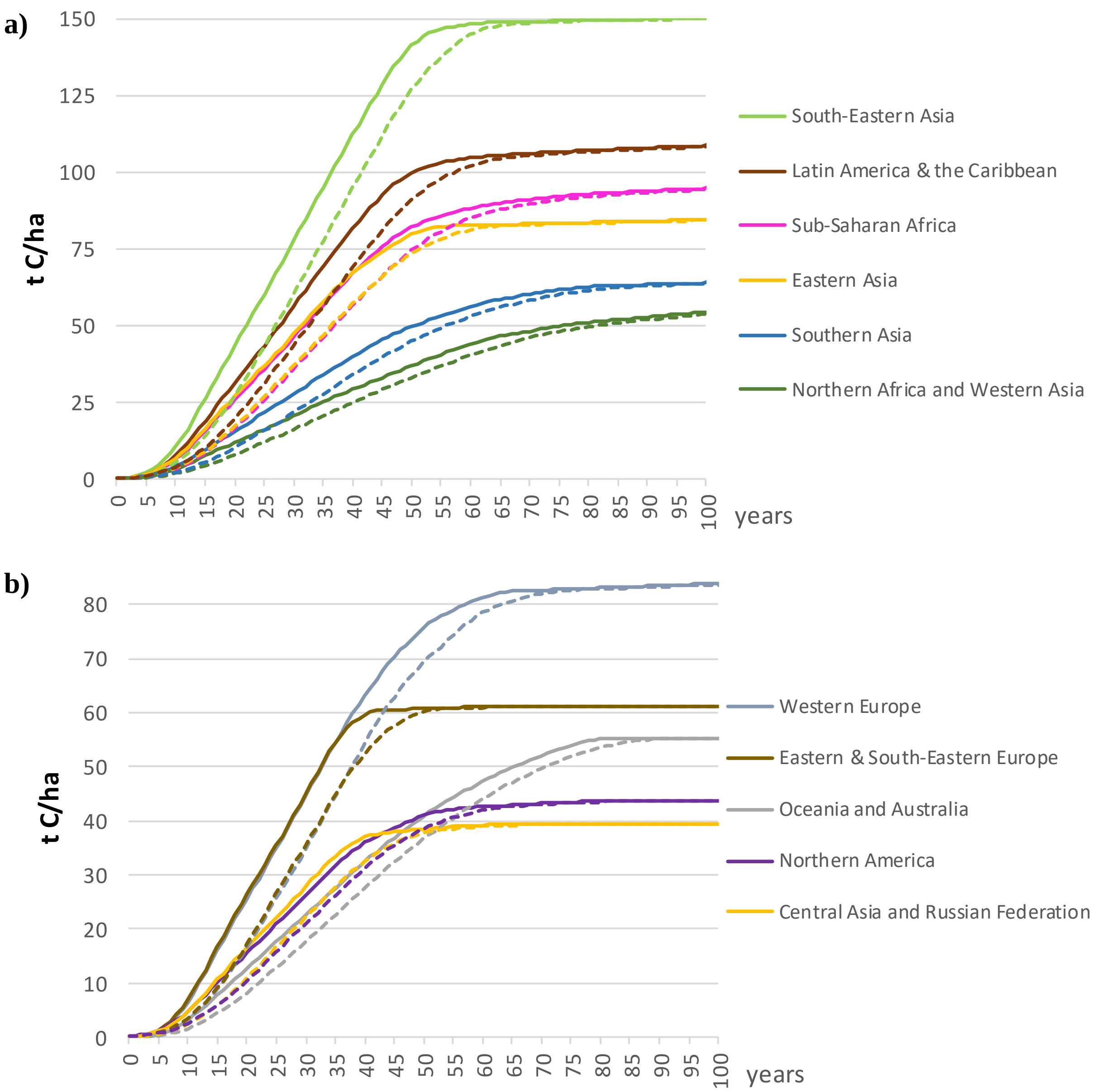

Figure S3. Carbon accumulation curves (including above- and belowground biomass) for world regions and transition periods of 10 years (solid lines) and 20 years (dashed lines); based on linear growth curves (see Fig. S6)
Source: own calculations based on default data from IPCC (2006b) and GIS data obtained from Erb et al. (2007), FAO (2012) and JRC (2018).

3 Energy plantation yields

The following figures show comparisons of the energy plantation yields assumed in this study with literature data. Our yield assumptions are based on potential net primary productivity (NPP_{pot}) and estimated losses. NPP_{pot} minus on-site losses is denoted "harvestable yield" in the figures. For the region "Northern Africa and Western Asia", the default SRC yield was adjusted to 5.6 tons of dry matter per hectare (based on Albanito et al., 2016), because NPP_{pot} minus losses was found to be an inadequate estimate for this world region.

Figure S4 shows a comparison with average yields assumed in assessments of global bioenergy potentials. Literature data for Europe, based on field trials, are compared with our assumptions for Europe in Figure S5. Albanito et al., (2016) used the “Lund-Potsdam-Jena managed land-dynamic global vegetation model” (LPJmL-DGVM) to derive biomass yields for short rotation energy plantations across continental and climatic regions (moist/dry, warm/cool). Figure S6 shows a comparison with our default assumptions.

The focus here is on relatively recent studies, for the following reason: Dickmann (2006, p. 703) notes that “early projections of yield tended to be overly optimistic because they were based on small plots that were managed more intensively than operational plantings could be and because pest depredations were not adequately accounted for” (cf. Hansen, 1991). According to Dickmann (2006, p. 703) “Realistic mean annual SRWC [short-rotation woody crops; note from the authors] increments generally fall within 5–20 Mg of biomass ha^{-1} yr^{-1} or 10–30 m^3 of wood ha^{-1} yr^{-1}, depending on species or clone, site, region, and cultural methods.”

To sum up, the average yields assumed in this study are well within the ranges of literature data, with a slight tendency towards the upper ranges. Since short rotation plantations are often assumed to be established on degraded land in potential assessments and field trials also include data from climatic regions with relatively little cropland, this tendency is considered appropriate.

Figure S7 shows a comparison of our default yields with maximum (NPPpot +20 %; 10 % losses) and minimum yields (NPP_{pot} – 20 %; 30 % losses) assumed in the sensitivity analysis.

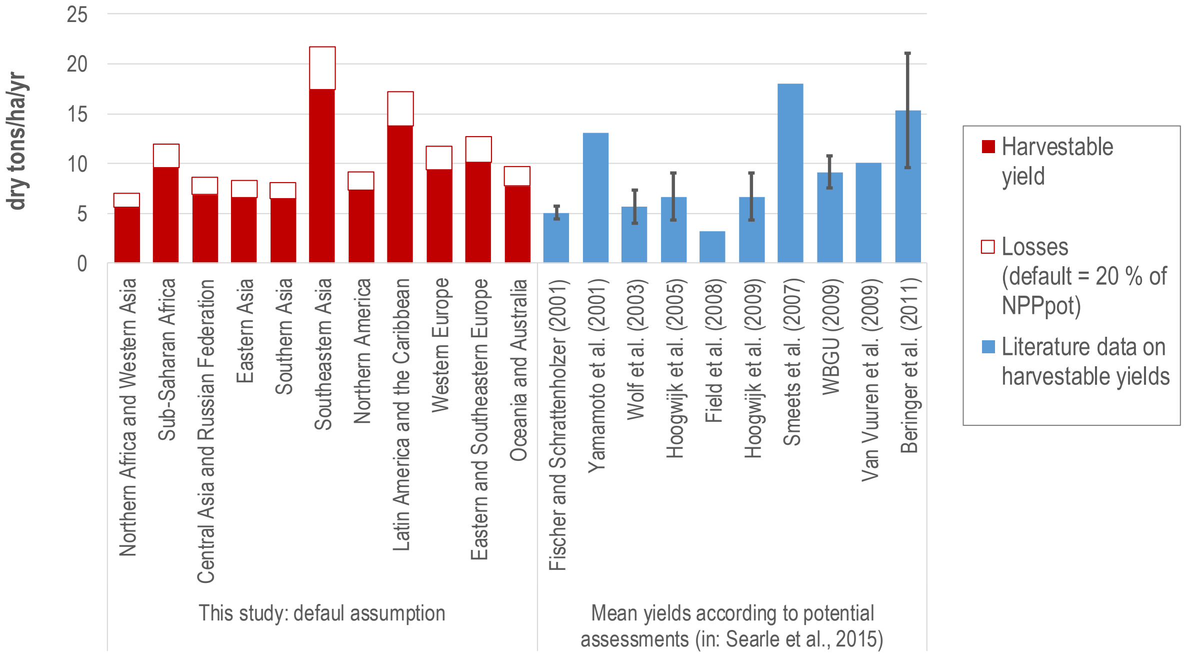

Figure S4. Yield of short rotation energy plantations: Comparison of default yields assumed in this study (harvestable yield and on-site losses) with mean harvestable yields assumed in studies on global bioenergy potentials

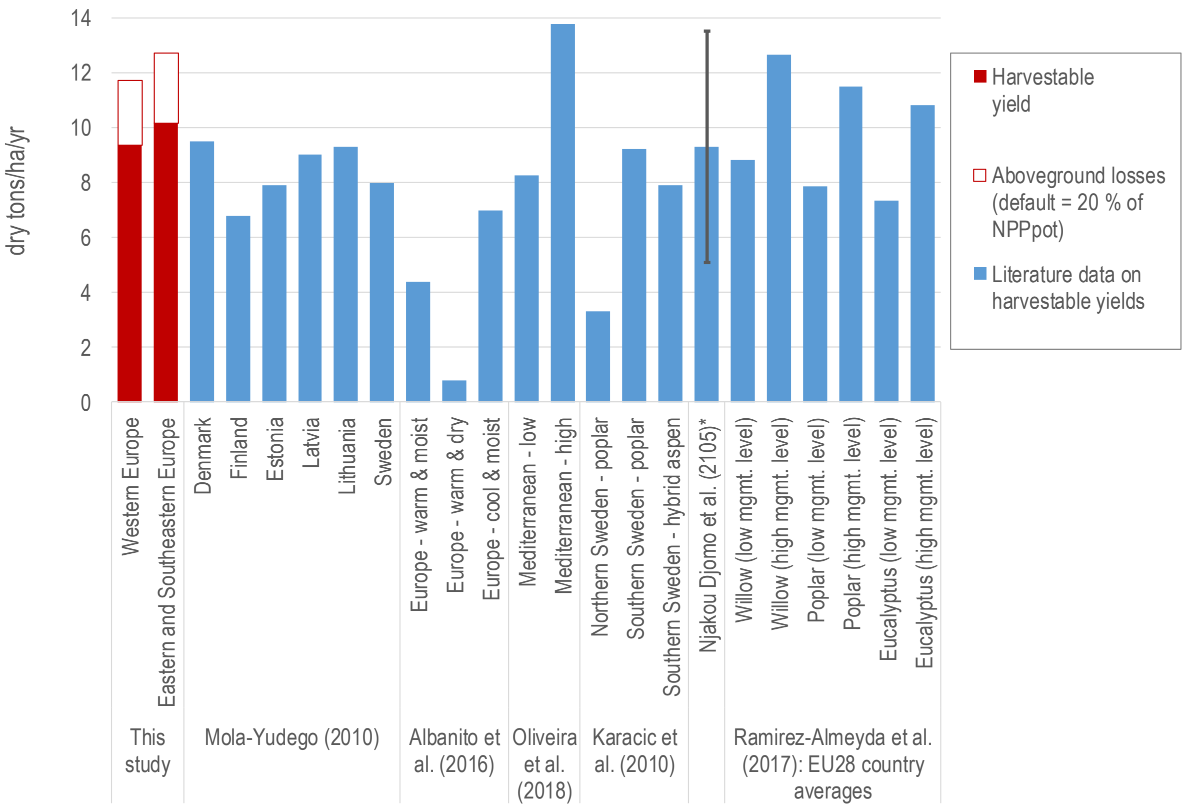

Figure S5. Yield of short rotation energy plantations in Europe: Comparison of default yields assumed in this study (harvestable yield and on-site losses) with literature data
**) Different species and sites within the EU*

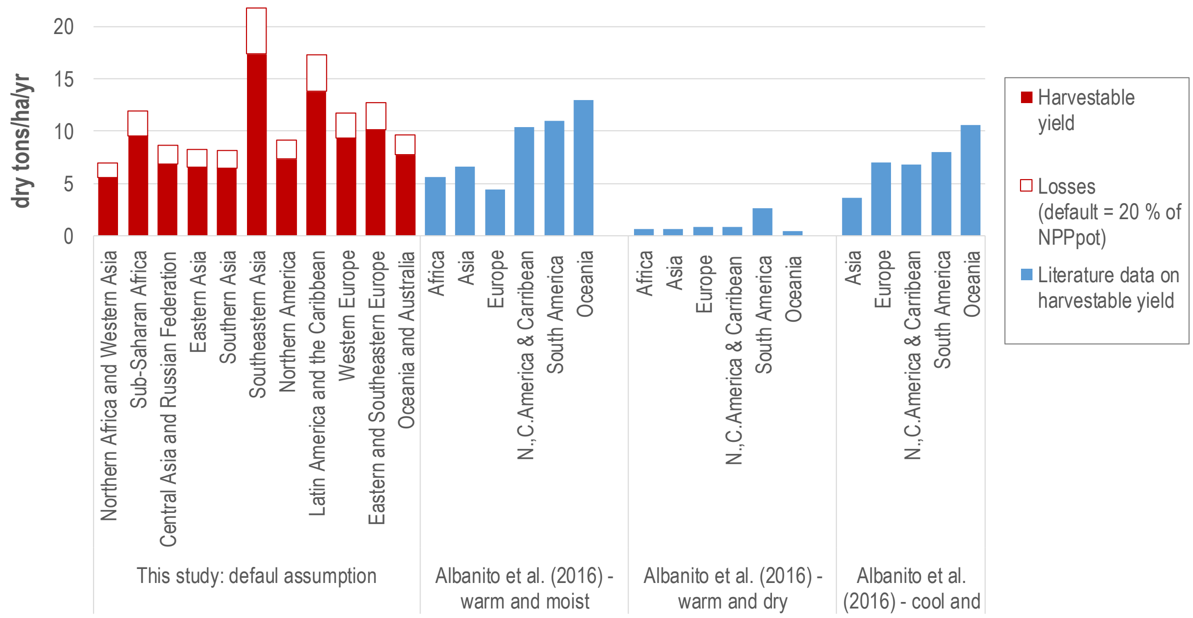

Figure S6. Yield of short rotation energy plantations: Comparison of default yields assumed in this study (harvestable yield and on-site losses) with climate- and continent specific values according to Albanito et al.; 2016)

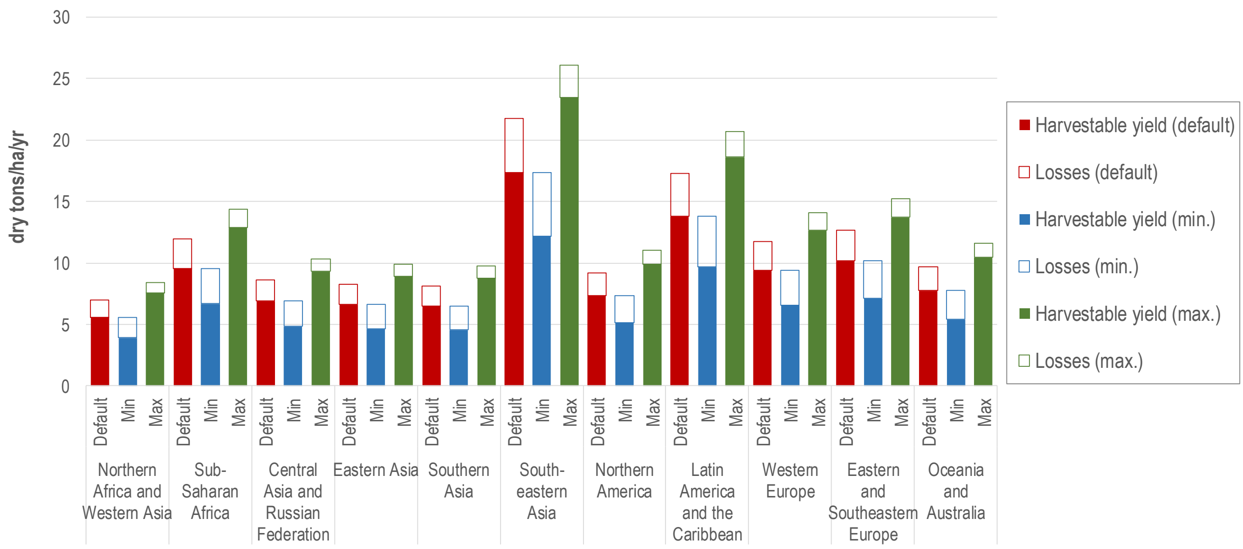

Figure S7. Yield of short rotation energy plantations: Comparison of default yields with maximum (NPPpot +20 %; 10 % on-site losses) and minimum yields (NPPpot – 20 %; 30 % on-site losses) assumed in the sensitivity analysis

4 Calculation of litter stocks in SRC

Litter stocks in energy plantations are calculated from on-site aboveground biomass losses. These losses $L_{on\text{-}site}$ are assumed to become litter and decay at constant decomposition rates k according to a first-order exponential function (Olson, 1963; Zhang, Hui, Luo, & Zhou, 2008):

$$s = \frac{S_t}{S_0} = e^{-k \cdot t}. \qquad (1)$$

s is the percent mass remaining at the elapsed time t, S_0 the initial litter stock and S_t the remaining litter stock at t. With constant litter inflows (Input), the change in litter stock is:

$$\frac{dS}{dt} = Input - k \cdot S \qquad (2)$$

The steady state (i.e. $\frac{dS}{dt} = 0$), corresponding to the final litter stock, is therefore calculated as

$$S = \frac{Input}{k} \qquad (3)$$

For our model, this means that the (world region-specific) C stock change in dead organic matter in case of land-use change from cropland to SRC corresponds to

$$\Delta C_{BE,wr}^{Litter} = \frac{L_{on-site}}{k_{wr}} \qquad (4)$$

with k_{wr} being world region-specific decay rates. Based on Zhang et al. (2008), it is assumed that decay rates in primarily tropic regions (South-Eastern Asia, Latin America and the Caribbean, Sub-Saharan Africa) are 1, and in other world regions 0.4. Since litter is the only considered fraction of dead organic matter under Tier 1, the change in dead organic matter C stocks corresponds to the change in litter C stocks:

$$\Delta C_{BE,wr}^{DOM} = \Delta C_{BE,wr}^{Litter} \tag{5}$$

5 Calculation of displacement factors

This section provides a description of the methods for calculating displacement factors. A glossary of symbols is provided in the Appendix.

5.1 Technologies with a single energy output

Displacement factors (DF) are calculated for different conversion technologies: heat production technologies (characterized by thermal efficiencies $\eta_{therm.}$) electricity generation plants (electrical efficiency; $\eta_{el.}$), combined heat and power plants (CHP; characterized by electrical and thermal efficiencies; $\eta_{el.}$ and $\eta_{therm.}$) and biofuel production plants (conversion efficiencies, $\eta_{conv.}$). For **technologies with a single energy output** (i.e. all types except for CHP plants[1]) and disregarding upstream emissions, the DF are calculated as

$$DF_{therm./el./conv.} = \frac{\eta_{therm./el./conv.,BE}}{\eta_{therm./el./conv.,fossil}} \cdot \frac{CE_{fossil}}{CE_{BM}} \tag{6}$$

With $CE_{fossil/BM}$ being the C emissions factors from the reference fossil fuel/woody biomass at combustion. The extended equation considering upstream emissions is provided in the main article.

5.2 Combined heat and power generation

In case of CHP, total fuel input is split into heat-related ($CE_{therm.}$) and electricity-related combustion emissions ($CE_{el.}$).

$$CE_{fossil/BM} = CE_{therm.,fossil/BM} + CE_{el.,fossil/BM} \tag{7}$$

Different allocation methods are in use for determining the shares attributed to heat and electricity (cf. Olsson, Wetterlund, & Söderström, 2015; Schüwer, Hanke, & Luhmann, 2016). The way, how the electricity-related shares $\varepsilon_{el.}$ are calculated have a strong impact on the DF. Here, two commonly used methods, the "Energy Allocation Method" and the "Finnish Method", are applied. These are considered to result in reasonable ranges of DF.

The **Energy Allocation Method** (Olsson et al., 2015; Fritsche & Schmidt, 2008); also called "IEA method" in (Mauch, Corradini, Wiesemeyer, & Schwentzek, 2010)) is based on the efficiency relations of the plant under consideration. The electricity allocation factors according to this method are:

$$\varepsilon_{el.,BE}^{EAM} = \frac{\eta_{el.,BE}}{\eta_{el.,BE}+\eta_{therm.,BE}} \tag{8}$$

[1] In fact, technological concepts for advanced biofuel plants often generate small amounts of surplus electricity (Ail & Dasappa, 2016; Hamelinck & Faaij, 2006), which are disregarded due to minor significance. In turn, the low estimates of conversions efficiencies are assumed 2 % higher than according values in literature.

$$\varepsilon_{el.,fossil}^{EAM} = \frac{\eta_{el.,ref}}{\eta_{el.,fossil} + \eta_{therm.,ref}} \tag{9}$$

The **DF for electricity from CHP** is then calculated as follows:

$$DF_{CHP}^{EAM} = \frac{\eta_{el.,BE}}{\eta_{el.,fossil}} \cdot \frac{CE_{fossil} \cdot \varepsilon_{el.,fossil}^{EAM}}{CE_{BE} \cdot \varepsilon_{el.,BE}^{EAM}} \tag{10}$$

The **Finnish Method** (Fritsche & Schmidt, 2008; Mauch et al., 2010) is based on the hypothetical primary energy savings (PES) provided by CHP according to the EU "Energy Efficiency Directive" (2012/27/EU):

$$PEE = 1 - \frac{1}{\eta_{therm.}/\eta_{therm.,sep} + \eta_{el.}/\eta_{el.,sep}} \tag{11}$$

$\eta_{el.,sep}$ and $\eta_{therm.,sep}$ are typical efficiencies of separate electricity and heat generation with the respective fuel. The allocation factor is then calculated as:

$$\varepsilon_{el.}^{Finn} = (1 - PEE) \cdot \frac{\eta_{el.}}{\eta_{el.,sep}} \tag{12}$$

Hence, the allocation factors for calculating DF for electricity from CHP plants with the Finnish Method are:

$$\varepsilon_{el.,BE}^{Finn} = \frac{\eta_{el.,BE}/\eta_{el.,BE,sep}}{\eta_{therm.,BE}/\eta_{therm.,BE,sep} + \eta_{el.,BE}/\eta_{el.,BE,sep}} \tag{13}$$

$$\varepsilon_{el.,fossil}^{Finn} = \frac{\eta_{el.,fossil}/\eta_{el.,fossil,sep}}{\eta_{therm.,fossil}/\eta_{therm.,fossil,sep} + \eta_{el.,fossil}/\eta_{el.,fossil,sep}} \tag{14}$$

6 Displacement factors and underlying data

6.1 Emission factors and upstream emissions

In the following table, emission factors for wood chips (from SCR) and fossil fuels as well as the assumed range of upstream emissions are presented.

Table S4. Emission factors and upstream emissions of fuel supply

Fuel type	Emission factors (CE) in g C/MJ	Upstream emissions (UE) in g $C_{eq.}$/MJ		Sources
		low	high	
Wood chips from SRC	30.5	2.2	6.8 (2.3)[a]	CE: IPCC (2006a), UE:Giuntoli et al. (2015), Schweier et al. (2017)
Anthracite (hard coal)	26.8	2.4	4.1	CE: IPCC (2006a), UE: Scull et al. (2017)
Natural gas	15.3	2.3	12.3	CE: IPCC (2006a), UE: Bradbury et al. (2013), EC (2015), Scull et al. (2017)
Diesel oil	20.2	1.1	9.5	CE: IPCC (2006a), UE: Brandt (2011), EC (2015)
Gasoline	18.9	1.1	9.5	CE: IPCC (2006a), UE: Brandt (2011), EC (2015)

Comments:

a) “High” values refer to long-distance overseas transport. The value in parenthesis is the high value assumed for liquid biofuel production. It is significantly lower than the one assumed for heat and power generation, because long-distance transport of feedstock for biofuel production is not considered plausible because transport as liquid biofuel is much more efficient.

6.2 Technology data

Technology data are summarized in the following tables. Efficiencies are based on the following references:

- **Heat generation**: Nian, Sun, Ma, & Li (2016); FNR (2007); Danish Energy Agency (2018); Kalt & Kranzl (2011); Gerssen-Gondelach et al. (2014).
- **Electricity generation**: Danish Energy Agency (2018); Cosijns & D’haeseleer (2007); Sims, Hastings, Schlamadinger, Taylor, & Smith (2006); Thornley et al. (2009); Wetterlund, Difs, & Söderström (2009); Wetterlund & Söderström (2010); IAMC (2018); Gerssen-Gondelach et al. (2014).
- **Liquid biofuel production**: Ail & Dasappa (2016); Hamelinck & Faaij (2006); Lynd et al. (2017); O’Connor (2013); Sims, Taylor, Saddler, & Mabee (2008); Daioglou et al. (2019); Gerssen-Gondelach et al. (2014).

Table S5. Technology data for bioenergy technologies

Category	No.	Description	$\eta_{therm.}$	$\eta_{el.}$	$\varepsilon_{el.,Finn.}$	$\varepsilon_{el.,EAM}$	PEE	$\eta_{conv.}$	C emissions per unit final energy (g C/MJ or g $C_{eq.}$/MJ)[a]		
									CE only	CE + UE (low)[b]	CE + UE (high)[c]
Heat	B1	Wood chip heat plant	85%	-	-	-	-	-	35.9	38.4	43.9
Electricity only (state-of-the-art)	B2	Wood chip steam plant	-	32%	-	-	-	-	95.3	102.1	116.6
Electricity only (2050)	B3	Wood chip BIGCC plant	-	42%	-	-	-	-	72.6	77.8	88.8
CHP (state-of-the-art)	B4	Wood chip steam plant	55%	26%	56%	32%	31%	-	65.3 / 37.7	70 / 40.3	79.9 / 46.1
CHP (2050)	B5	Wood chip BIGCC CHP plant	44%	40%	71%	48%	43%	-	49.4 / 36.3	52.9 / 38.9	60.4 / 44.4
Liquid biofuels (2050)	B6	BtL diesel (FT synthesis) - pessimistic	-	-	-	-	-	30%	101.7	108.9	110.7
	B7	BtL diesel (FT synthesis) - optimistic	-	-	-	-	-	55%	55.5	59.4	60.4
	B8	Cellulosic ethanol -pessimistic	-	-	-	-	-	35%	82.4	88.3	89.8
	B9	Cellulosic ethanol - optimistic	-	-	-	-	-	51%	58.7	62.8	63.9

Comments:

a) Upstream emissions are given in g C equivalent (g $C_{eq.}$) per MJ. For CHP: electricity-related emissions only (Allocation method: Finnish/Energy allocation).

b) Basis for calculating "favourable BE scenarios" in Fig. 3.

c) Basis for calculating "unfavourable BE scenarios" in Fig. 3.

Table S6. Technology data for fossil fuel-based reference technologies

Category	No.	Description	$\eta_{therm.}$	$\eta_{el.}$	$\varepsilon_{el.,Finn.}$	$\varepsilon_{el.,EAM}$	PEE	$\eta_{conv.}$	C emissions per unit final energy (g C/MJ or g $C_{eq.}$/MJ)[a]		
									CE only	CE + UE (low)[b]	CE + UE (high)[c]
Heat	F1	Natural gas heat plant	90%	-	-	-	-	-	17.0	19.6	30.6
	F2	Hard coal heat plant	90%	-	-	-	-	-	29.8	32.5	34.3
	F3	Oil heat plant	90%	-	-	-	-	-	22.4	23.7	33.0
Electricity only (state-of-the-art)	F4	Natural gas CCGT plant	-	58%	-	-	-	-	26.4	30.4	47.5
	F5	Coal condensing plant (PCC)	-	44%	-	-	-	-	60.9	66.4	70.2
Electricity only (2050)	F6	Natural gas CCGT plant	-	62%	-	-	-	-	24.7	28.4	44.5
	F7	Coal condensing plant (PCC)	-	46%	-	-	-	-	58.3	63.5	67.1
	F8	Coal IGCC plant	-	48%	-	-	-	-	55.8	60.9	64.3
CHP (state-of-the-art)	F9	Natural gas CCGT CHP plant	45%	45%	61%	50%	22%	-	20.7 / 17	23.8 / 19.6	37.2 / 30.6
	F10	Coal CHP plant (PCC)	42%	44%	69%	51%	31%	-	42.2 / 31.2	46 / 34	48.6 / 35.9
CHP (2050)	F11	Natural gas CCGT CHP plant	47%	45%	58%	49%	21%	-	19.6 / 16.6	22.6 / 19.1	35.3 / 30
	F12	Coal CHP plant (PCC)	44%	44%	66%	50%	31%	-	40.3 / 30.5	44 / 33.2	46.5 / 35.1
Liquid transport fuels (2050)	F13	Diesel oil	-	-	-	-	-	94%[d]	21.5	22.6	31.6
	F14	Gasoline	-	-	-	-	-	94%[d]	20.1	21.3	30.3

Comments:

a) Upstream emissions are given in g C equivalent (g $C_{eq.}$) per MJ. For CHP: electricity-related emissions only (Allocation method: Finnish/Energy allocation).

b) Basis for calculating "unfavourable BE scenarios" in Fig. 3.

c) Basis for calculating "favourable BE scenarios" in Fig. 3.

d) For fossil diesel and gasoline, a conversion efficiency of 94 % is assumed based on the assumption of 6 % refinery own consumption and losses (Eurostat, 2018)

6.3 Displacement factors

Table S7. Displacement factors of bioenergy technologies (data to Fig. 3b)

Fossil fuel-based tech.	Biomass-based counterpart	Converions route	Fossil fuel displaced[a]	Displacement factors[b]: Upstream emissions excluded	Displacement factors[b]: Incl. upstream emi. (favourable BE scenario)	Displacement factors[b]: Incl. upstream emi. (unfavourable BE scenario)
F1	B1	Heat generation	NG	0.47	0.78	0.32
F2	B1		HC	0.83	0.88	0.68
F3	B1		PD	0.63	0.85	0.44
F4	B2	Electricity only (state-of-the-art)	NG	0.28	0.43	0.10
F5	B2		HC	0.64	0.67	0.47
F6	B3	Electricity only (2050)	NG	0.34	0.54	0.17
F7	B3		HC	0.80	0.85	0.65
F8	B3		HC	0.77	0.81	0.62
F9	B4	Electricity from CHP (state-of-the-art)	NG	0.32 / 0.45	0.5 / 0.74	0.14 / 0.3
F10	B4		HC	0.65 / 0.83	0.67 / 0.88	0.48 / 0.68
F11	B5	Electricity from CHP (2050)	NG	0.4 / 0.46	0.64 / 0.75	0.23 / 0.3
F12	B5		HC	0.82 / 0.84	0.87 / 0.9	0.67 / 0.69
F13	B6	Transport fuels (opt.)	PD	0.21	0.24	0.13
F13	B7	Transport fuels (pess.)	PD	0.39	0.50	0.32
F14	B8	Transport fuels (opt.)	PD	0.24	0.30	0.17
F14	B9	Transport fuels (pess.)	PD	0.34	0.44	0.27

Comments:

a) NG: natural gas; HC: hard coal; PD: Petroleum derivatives

b) Displacement factors for F9 to F12 are based on Finnish/Energy allocation method

7 Full results to the sensitivity analysis regarding yields and losses

Figure S8 shows the complete results of the sensitivity analysis regarding SRC yields and on-site losses.

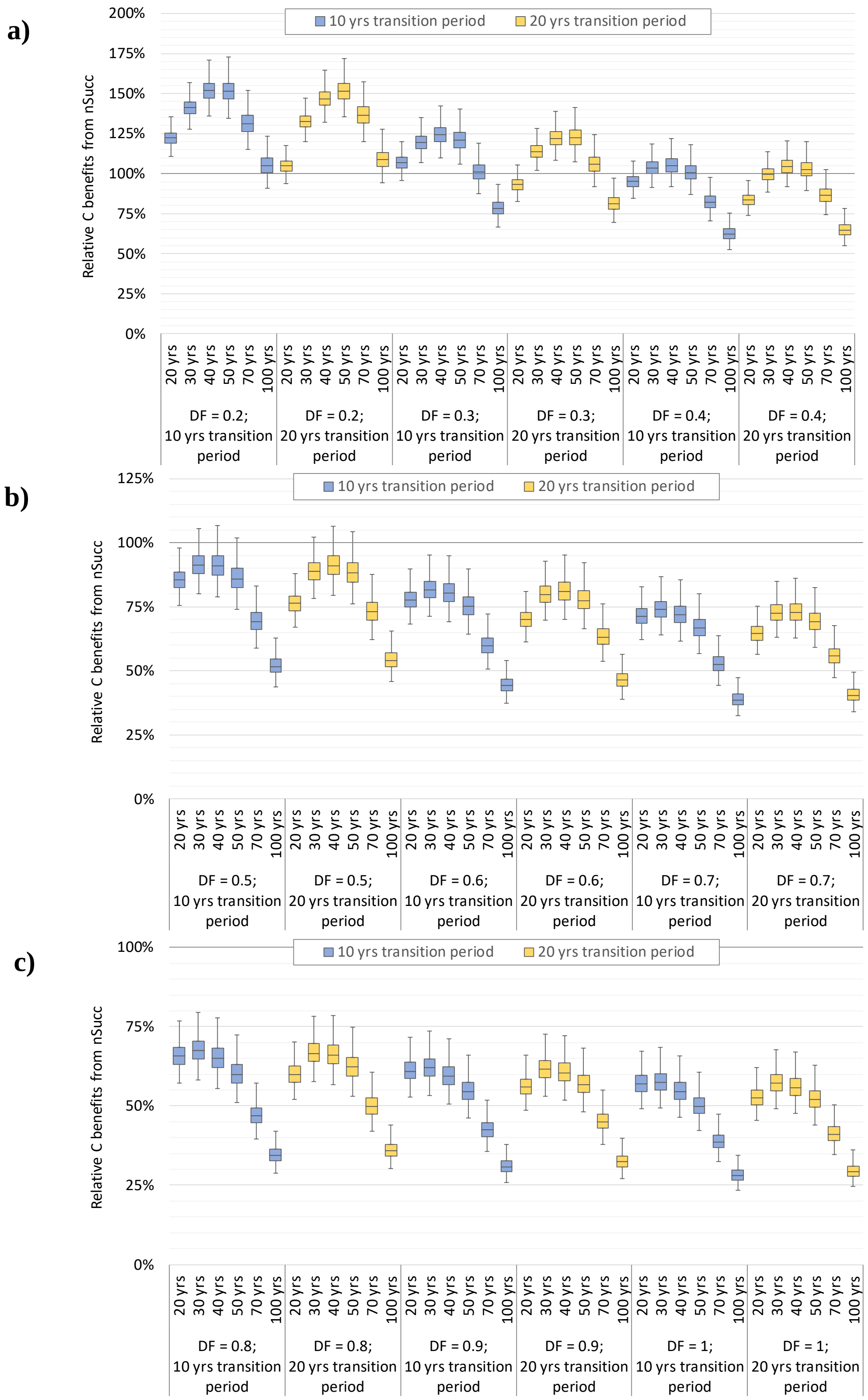

Figure S8. Sensitivity analysis regarding energy plantation yields (+/- 30 %) and losses (+/- 10 %, i.e. 10 to 30 % of annual biomass growth): Results from Monte-Carlo simulations assuming uniform distribution in these ranges and DF ranging from 0.2 to 0.4 (a), 0.5 to 0.7 (b) and 0.8 to 1.0 (c).

Appendix: Glossary to section 5

DF..............................Displacement factor

$\eta_{therm.}$ ....Thermal efficiency

$\eta_{el.}$ ....Electrical efficiency

$\eta_{conv.}$ ....Conversion efficiency

$\varepsilon_{el.}$...............................Allocation factor for electricity (share of the total emissions allocated to electricity generation in CHP plants)

CE..............................Carbon emission factors

PES............................Primary energy savings

Further subscripts:

BE..............................Bioenergy technology

BM.............................Biomass fuel (wood biomass)

$fossil$..........................Fossil fuel or fossil fuel-based (technology)

sep.............................separate (heat and power generation; in contrast to CHP)

Superscripts (indicating allocation methods):

EAM................... Energy Allocation Method

$Finn$................... Finnish Method

Literature

Ail, S. S., & Dasappa, S. (2016). Biomass to liquid transportation fuel via Fischer Tropsch synthesis – Technology review and current scenario. Renewable and Sustainable Energy Reviews, 58, 267–286. https://doi.org/10.1016/j.rser.2015.12.143

Albanito, F., Beringer, T., Corstanje, R., Poulter, B., Stephenson, A., Zawadzka, J., & Smith, P. (2016). Carbon implications of converting cropland to bioenergy crops or forest for climate mitigation: a global assessment. GCB Bioenergy, 8(1), 81–95. https://doi.org/10.1111/gcbb.12242

Beringer, T., Lucht, W., & Schaphoff, S. (2011). Bioenergy production potential of global biomass plantations under environmental and agricultural constraints. GCB Bioenergy, 3(4), 299–312. https://doi.org/10.1111/j.1757-1707.2010.01088.x

Bradbury, J., Obeiter, M., Draucker, L., Wang, W., & Stevens, A. (2013). Clearing the air: Reducing upstream greenhouse gas emissions from U.S. natural gas systems (Working Paper) (p. 60). Washington, DC: World Resources Institute. Retrieved from http://www.wri.org/publication/ clearing-the-air

Brandt, A. R. (2011). Upstream greenhouse gas (GHG) emissions from Canadian oil sands as a feedstock for European refineries. Department of Energy Resources Engineering, Stanford University.

Cosijns, L., & D'haeseleer, W. (2007). EUSUSTEL - European Sustainable Electricity; Comprehensive Analysis of Future European Demand and Generation of European Electricity and its Security of Supply. Final Technical Report.

Daioglou, V., Doelman, J. C., Wicke, B., Faaij, A., & van Vuuren, D. P. (2019). Integrated assessment of biomass supply and demand in climate change mitigation scenarios. Global Environmental Change, 54, 88–101. https://doi.org/10.1016/j.gloenvcha.2018.11.012

Danish Energy Agency. (2018). Technology data for energy plants for electricity and district heating generation.

Dickmann, D. (2006). Silviculture and biology of short-rotation woody crops in temperate regions: Then and now. Biomass and Bioenergy, 30(8–9), 696–705. https://doi.org/10.1016/j.biombioe.2005.02.008

EC. (2015). Study on actual GHG data for diesel, petrol, kerosene and natural gas. Final report. European Commission, DG ENER.

Erb, K.-H., Gaube, V., Krausmann, F., Plutzar, C., Bondeau, A., & Haberl, H. (2007). A comprehensive global 5 min resolution land-use data set for the year 2000 consistent with national census data. Journal of Land Use Science, 2(3), 191–224. https://doi.org/10.1080/17474230701622981

Eurostat (2018). Energy balance sheets – 2014 data – 2016 edition. Retrieved from https://ec.europa.eu/eurostat/web/products-statistical-books/-/KS-EN-16-001

FAO. (2012). Global ecological zones for FAO forest reporting: 2010 Update (Forest Resources Assessment Working Paper 179). Rome: Food and Agriculture Organisation of the United Nations.

Field CB, Campbell JE, Lobell DB (2008) Biomass energy: the scale of the potential resource. Trends in ecology & evolution, 23, 65–72.

Fischer, G., & Schrattenholzer, L. (2001). Global bioenergy potentials through 2050. Biomass and Bioenergy, 9.

FNR. Leitfaden Bioenergie – Planung, Betrieb und Wirtschaftlichkeit von Bioenergieanlagen, 3. Auflage 2007, Fachagentur Nachwachsende Rohstoffe e.V. (FNR), Gülzow; 2007.

Fritsche, U., & Rausch, L. (2008). Bestimmung spezifischer Treibhausgas-Emissionsfaktoren für Fernwärme (No. Forschungsbericht 360 16 008). Dessau-Roßlau: Öko-Institut.

Fritsche, U., & Schmidt, K. (2008). Global Emission Model of Integrated Systems (GEMIS). Manual. Darmstadt: Öko-Institut (Institute for Applied Ecology).

Gabrielle, B., The, N. N., Maupu, P., & Vial, E. (2013). Life cycle assessment of eucalyptus short rotation coppices for bioenergy production in southern France. GCB Bioenergy, 5(1), 30–42. https://doi.org/10.1111/gcbb.12008

Gerssen-Gondelach, S. J., Saygin, D., Wicke, B., Patel, M. K., & Faaij, A. P. C. (2014). Competing uses of biomass: Assessment and comparison of the performance of bio-based heat, power, fuels and materials. Renewable and Sustainable Energy Reviews, 40, 964–998. https://doi.org/10.1016/j.rser.2014.07.197

Giuntoli, J., Agostini, A., Edwards, R., Marelli, L., & et al. (2015). Solid and gaseous bioenergy pathways: input values and GHG emissions: calculated according to the methodology set in COM(2010) 11 and SWD(2014) 259. Luxembourg: Publications Office. Retrieved from http://bookshop.europa.eu/uri?target=EUB:NOTICE:LDNA27215:EN:HTML

Hamelinck, C. N., & Faaij, A. P. C. (2006). Outlook for advanced biofuels. Energy Policy, 34(17), 3268–3283.

Hansen EA. Poplar woody biomass yields: a look to the future. Biomass Bioenergy 1991;1:1–7.

Hoogwijk M, Faaij A, De Vries B, Turkenburg W (2009) Exploration of regional and global cost–supply curves of biomass energy from short-rotation crops at abandoned cropland and rest land under four IPCC SRES land-use scenarios. Biomass and Bioenergy, 33, 26–43.

Hoogwijk, M., Faaij, A., Eickhout, B., Devries, B., & Turkenburg, W. (2005). Potential of biomass energy out to 2100, for four IPCC SRES land-use scenarios. Biomass and Bioenergy, 29(4), 225–257. https://doi.org/10.1016/j.biombioe.2005.05.002

IAMC (2018) IAMC wiki. The common Integrated Assessment Model (IAM) documentation. Retrieved from: http://iamcdocumentation.eu/index.php/IAMC_wiki, last access in Sept., 2018

IPCC. (2006a). 2006 IPCC Guidelines for National Greenhouse Gas Inventories. Volume 2: Energy (Prepared by the National Greenhouse Gas Inventories Programme). IGES, Japan.

IPCC. (2006b). 2006 IPCC Guidelines for National Greenhouse Gas Inventories. Volume 4: Agriculture, Forestry and Other Land Use (Prepared by the National Greenhouse Gas Inventories Programme). IGES, Japan.

JRC. (2018). Renewable Energy Directive. Thematic Data Layers for Commission Decision of [10 June 2010] on guidelines for the calculation of land carbon stocks for the purpose of Annex V to Directive 2009/28/EC. Joint Research Centre of the European Commission. Retrieved from https://esdac.jrc.ec.europa.eu/projects/renewable-energy-directive

Kalt, G., & Kranzl, L. (2011). Assessing the economic efficiency of bioenergy technologies in climate mitigation and fossil fuel replacement in Austria using a techno-economic approach. Applied Energy, 88(11), 3665–3684.

Karacic, A., Verwijst, T., & Weih, M. (2003). Above-ground Woody Biomass Production of Short-rotation Populus Plantations on Agricultural Land in Sweden. Scandinavian Journal of Forest Research, 18(5), 427–437. https://doi.org/10.1080/02827580310009113

Lynd, L. R., Liang, X., Biddy, M. J., Allee, A., Cai, H., Foust, T., … Wyman, C. E. (2017). Cellulosic ethanol: status and innovation. Current Opinion in Biotechnology, 45, 202–211. https://doi.org/10.1016/j.copbio.2017.03.008

Mauch, W., Corradini, R., Wiesemeyer, K., & Schwentzek, M. (2010). Allokationsmethoden für spezifische CO2-Emissionen von Strom und Wärme aus KWK-Anlagen. Energiewirtschaftliche Tagesfragen, 55(9).

Mola-Yudego, B. (2010). Regional potential yields of short rotation willow plantations on agricultural land in Northern Europe. Silva Fennica, 44(1). https://doi.org/10.14214/sf.163

Nian, V., Sun, Q., Ma, Z., & Li, H. (2016). A Comparative Cost Assessment of Energy Production from Central Heating Plant or Combined Heat and Power Plant. Energy Procedia, 104, 556–561. https://doi.org/10.1016/j.egypro.2016.12.094

O’Connor, D. (2013). Advanced Biofuels–GHG Emissions and Energy Balances. Report IEA Bioenergy Task 39. Retrieved from http://task39.org/files/2013/05/Energy-and-GHG-Emissions-IEA-Bioenergy-T39-Report-May-2013-rev.pdf

Oliveira, N., Rodríguez-Soalleiro, R., Pérez-Cruzado, C., Cañellas, I., Sixto, H., & Ceulemans, R. (2018). Above- and below-ground carbon accumulation and biomass allocation in poplar short rotation plantations under Mediterranean conditions. Forest Ecology and Management, 428, 57–65. https://doi.org/10.1016/j.foreco.2018.06.031

Olson, J. S. (1963). Energy Storage and the Balance of Producers and Decomposers in Ecological Systems. Ecology, 44(2), 322–331. https://doi.org/10.2307/1932179

Olsson, L., Wetterlund, E., & Söderström, M. (2015). Assessing the climate impact of district heating systems with combined heat and power production and industrial excess heat. Resources, Conservation and Recycling, 96, 31–39. https://doi.org/10.1016/j.resconrec.2015.01.006

Prestele, R., Hirsch, A. L., Davin, E. L., Seneviratne, S. I., & Verburg, P. H. (2018). A spatially explicit representation of conservation agriculture for application in global change studies. Global Change Biology. https://doi.org/10.1111/gcb.14307

Ramirez-Almeyda, J., Elbersen, B., Monti, A., Staritsky, I., Panoutsou, C., Alexopoulou, E., … Elbersen, W. (2017). Assessing the Potentials for Nonfood Crops. In Modeling and Optimization of Biomass Supply Chains (pp. 219–251). Elsevier. https://doi.org/10.1016/B978-0-12-812303-4.00009-4

Schüwer, D., Hanke, T., & Luhmann, H.-J. (2016). Konsistenz und Aussagefähigkeit der Primärenergie-Faktoren für Endenergieträger im Rahmen der EnEV. Retrieved from https://www.recknagel-online.de/fileadmin/Recknagel/Nachrichten/bericht-primaerenergiefaktoren-pef-enev.pdf

Schweier, J., Molina-Herrera, S., Ghirardo, A., Grote, R., Díaz-Pinés, E., Kreuzwieser, J., … Becker, G. (2017). Environmental impacts of bioenergy wood production from poplar short-rotation coppice grown at a marginal agricultural site in Germany. GCB Bioenergy, 9(7), 1207–1221. https://doi.org/10.1111/gcbb.12423

Scull, B. D., Kaddoura, S., Chen, K., Gyourgis, N., Liu, Y., Miller, S. G., … Yan, E. (2017). Upstream Emissions of Coal and Gas. New York: NY: Columbia University, School of International and Public Affairs.

Searle, S., & Malins, C. (2015). A reassessment of global bioenergy potential in 2050. GCB Bioenergy, 7(2), 328–336. https://doi.org/10.1111/gcbb.12141

Sims, R. E. H., Hastings, A., Schlamadinger, B., Taylor, G., & Smith, P. (2006). Energy crops: current status and future prospects. Global Change Biology, 12(11), 2054–2076. https://doi.org/10.1111/j.1365-2486.2006.01163.x

Sims, R., Taylor, M., Saddler, J., & Mabee, W. (2008). From 1st to 2nd generation biofuel technologies. An overview of current industry and RD&D activities. OECD/IEA, IEA Bioenergy.

Smeets, E. M. W., Faaij, A. P. C., Lewandowski, I. M., & Turkenburg, W. C. (2007). A bottom-up assessment and review of global bio-energy potentials to 2050. Progress in Energy and Combustion Science, 33(1), 56–106.

Thornley, P., Upham, P., Huang, Y., Rezvani, S., Brammer, J., & Rogers, J. (2009). Integrated assessment of bioelectricity technology options. Energy Policy, 37(3), 890–903.

Van Vuuren, D. P., Van Vliet, J., & Stehfest, E. (2009). Future bio-energy potential under various natural constraints. Energy Policy, 37(11), 4220–4230.

WBGU (Ed.). (1998). The Accounting of Biological Sinks and Sources Under the Kyoto Protocol: A Step Forwards or Backwards for Global Environmental Protection? Bremerhaven.

Wetterlund, E., & Söderström, M. (2010). Biomass gasification in district heating systems – The effect of economic energy policies. Applied Energy, 87(9), 2914–2922. https://doi.org/10.1016/j.apenergy.2009.11.032

Wetterlund, E., Difs, K., & Söderström, M. (2009). Energy policies affecting biomass gasification applications in district heating systems. In Hong Kong. Hong Kong.

Winrock International. (2014). AFOLU Carbon Calculator. The afforestation/reforestation tool: Underlying data and methods. US Aid.

Wolf J, Bindraban P, Luijten J, Vleeshouwers L (2003) Exploratory study on the land area required for global food supply and the potential global production of bioenergy. Agricultural Systems, 76, 841–861.

Yamamoto, H., Fujino, J., & Yamaji, K. (2001). Evaluation of bioenergy potential with a multi-regional global-land-use-and-energy model. Biomass and Bioenergy, 21(3), 185–203. https://doi.org/10.1016/S0961-9534(01)00025-3

Zhang, D., Hui, D., Luo, Y., & Zhou, G. (2008). Rates of litter decomposition in terrestrial ecosystems: global patterns and controlling factors. Journal of Plant Ecology, 1(2), 85–93. https://doi.org/10.1093/jpe/rtn002
